# Bacterial and Chemical Evidence of Coastal Water Pollution from the Tijuana River in Sea Spray Aerosol

**DOI:** 10.1021/acs.est.2c02312

**Published:** 2023-03-02

**Authors:** Matthew A. Pendergraft, Pedro Belda-Ferre, Daniel Petras, Clare K. Morris, Brock A. Mitts, Allegra T. Aron, MacKenzie Bryant, Tara Schwartz, Gail Ackermann, Greg Humphrey, Ethan Kaandorp, Pieter C. Dorrestein, Rob Knight, Kimberly A. Prather

**Affiliations:** † Scripps Institution of Oceanography, University of California San Diego, San Diego, La Jolla, California 92037, United States; ‡ Department of Pediatrics, University of California, San Diego, La Jolla, California 92093, United States; § Collaborative Mass Spectrometry Innovation Center, Skaggs School of Pharmacy and Pharmaceutical Science, University of California, San Diego, La Jolla, California 92093, United States; ∥ CMFI Cluster of Excellence, Interfaculty Institute of Microbiology and Medicine, University of Tuebingen, Tuebingen 72076, Germany; ⊥ Department of Chemistry and Biochemistry, University of California, San Diego, La Jolla, California 92093, United States; # Independent Researcher, Darwin, California 93522, United States; ¶ Center for Microbiome Innovation, University of California, San Diego, La Jolla, California 92093, United States; ∇ Department of Bioengineering, University of California, San Diego, La Jolla, California 92093, United States; ○ Department of Computer Sciences and Engineering, University of California, San Diego, La Jolla, California 92093, United States; ⬢ Department of Chemistry and Biochemistry, University of Denver, Denver, Colorado 80210, United States

**Keywords:** water pollution, coastal, sea spray aerosol, pathogen, airborne exposure, 16S, mass spectrometry, Imperial Beach, Tijuana, Tijuana River, Scripps Institution of Oceanography

## Abstract

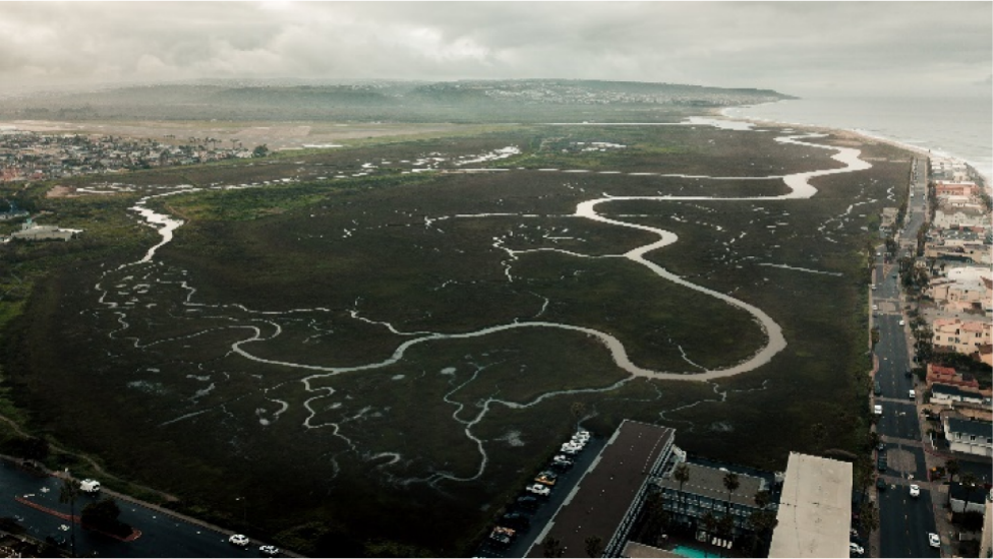

Roughly half of the human population lives near the coast, and coastal water pollution (CWP) is widespread. Coastal waters along Tijuana, Mexico, and Imperial Beach (IB), USA, are frequently polluted by millions of gallons of untreated sewage and stormwater runoff. Entering coastal waters causes over 100 million global annual illnesses, but CWP has the potential to reach many more people on land *via* transfer in sea spray aerosol (SSA). Using 16S rRNA gene amplicon sequencing, we found sewage-associated bacteria in the polluted Tijuana River flowing into coastal waters and returning to land in marine aerosol. Tentative chemical identification from non-targeted tandem mass spectrometry identified anthropogenic compounds as chemical indicators of aerosolized CWP, but they were ubiquitous and present at highest concentrations in continental aerosol. Bacteria were better tracers of airborne CWP, and 40 tracer bacteria comprised up to 76% of the bacteria community in IB air. These findings confirm that CWP transfers in SSA and exposes many people along the coast. Climate change may exacerbate CWP with more extreme storms, and our findings call for minimizing CWP and investigating the health effects of airborne exposure.

## Abstract 2

Here, we use 16S amplicon sequencing and tandem mass spectrometry to identify bacteria and chemicals in the polluted Tijuana River flowing into coastal waters and transferring to the coastal atmosphere in sea spray aerosol.

## Introduction

Coastal water pollution (CWP) is an ever-growing global environmental problem and public health threat. Over one hundred thousand cases of illness and tens of thousands of deaths occur annually worldwide due to people entering contaminated waters or eating tainted seafood.^1^ Swimming and surfing in polluted waters increase the incidence of multiple types of illness.^2,3^ Untreated sewage and stormwater runoff are common causes of CWP. Oils, fuels, metals, plastics, drugs, insecticides, detergents, solvents, and fire retardants are common chemical contaminants.^4,5^*Escherichia coli* (*E. coli*) and *Enterococcus* spp. are bacteria used as sewage indicators, whereas enteroviruses, human norovirus, hepatitis A virus, and SARS-CoV2 are actual pathogens found in sewage-contaminated waters.^6−8,69^ Pathogens in coastal waters pose an immediate health threat because illness can occur from a single exposure.^1,6^

CWP at the Mexico–USA border between Tijuana (TJ), Mexico, and Imperial Beach (IB), USA, has persisted for decades and has been officially declared a state of emergency.^7,9,11^ Whereas fecal and chemical pollution from stormwater runoff has been detected at various beaches in San Diego (SD), there is persistent and severe CWP at IB and TJ.^5,7,10,11^ Rains and inadequate infrastructure result in untreated sewage flowing into TJ–IB coastal waters. Hepatitis A virus and bacteria from TJ sewage have been detected in IB coastal waters.^7,12^ The Tijuana River (TJR) is a major pollution conduit that sends 100-million-gallon sewage spills into South IB coastal waters.^12,13,72^ SARS-CoV-2 has been detected in TJR waters at concentrations matching those at wastewater treatment plants.^69^ These problems caused IB beaches to be closed to water contact for 295 days 81 in 2020.^14^ This problem will likely persist after implementation of planned infrastructure due to multiple sources and continued diversion of high flow stormwater and sewage directly to the ocean.^13^ Climate change is expected to cause more extreme precipitation events, which may further exacerbate the problem.^15^

CWP has the potential to transfer to the atmosphere in sea spray aerosol (SSA) and reach people on land through airborne transport.^16^ SSA is formed by breaking waves and bursting bubbles that eject microscopic seawater aerosol into the atmosphere, ranging in size from tens of nanometers to tens of microns.^17^ SSA contains diverse chemical compounds and microorganisms from the source waters, including bacteria (∼1 μm diameter) and viruses (∼0.1 μm diameter).^18,19^ Microbes and chemical compounds can become greatly enriched in SSA by bubble scavenging and bursting through the ocean’s surface microlayer.^20−22^ Once in the atmosphere, SSA can travel hundreds of kilometers.^17^ Prior research used a tracer dye to demonstrate the transfer of CWP in SSA.^16^ Here, we present evidence from non-targeted tandem mass spectrometry and 16S amplicon sequencing of CWP transferring to the atmosphere in SSA.

## Materials and Methods

### Sampling

**Figure 1:**
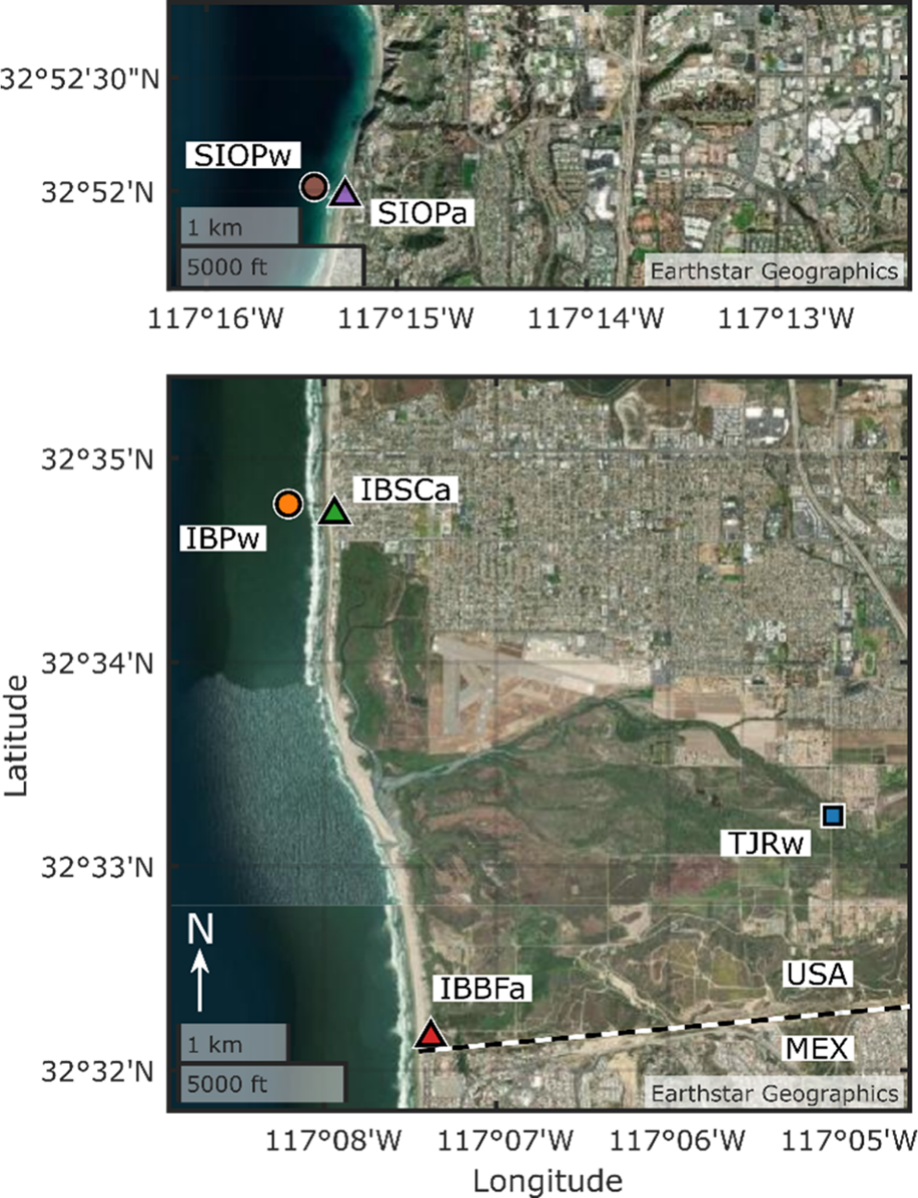
Site map and sampling locations. Displayed are the locations of aerosol and water sampling at IB, CA, USA (bottom) and 35 km away at SIO in La Jolla, CA, USA (top). Marker formatting is consistent in all figures. The dashed line denotes the Mexico–USA border. Sites of water sampling are denoted with a “w”, and sites of aerosol sampling are denoted with an “a”. Produced using MATLAB version 9.10.0.1602886 (R2021a).^37^ Map imagery reprinted with permission from Earthstar Geographics. Copyright 2022 Earthstar Geographics/Terracolor.

To investigate the airborne transport of CWP, we sampled coastal water and aerosol in IB and at Scripps Institution of Oceanography (SIO) in five sampling rounds (SRs) following rain events from January to May of 2019 (Figures 1 and S1). We chose IB for its frequent and severe water quality issues, and SIO served as a reference site. Coastal water quality at both IB and SIO can be impacted by stormwater runoff, so by comparing IB to SIO, we investigate signs of CWP that are above common levels for the region.^10^ We collected coastal water daily from the West (seaward) ends of the IB and SIO piers (IBPw and SIOPw, SR 1-5, Figure 1) into acid-cleaned buckets and bottles. CWP is transported by ocean currents and is a challenge to sample.^23^ We overcame this by directly sampling the TJR (TJRw in Figure 1; SR 2-5), which was actively flowing into IB coastal waters throughout the study (Figure S1) and is a major pathway of CWP in the area.^12,69,72^

Coastal aerosol was sampled at one location each day in IB during SR 1-4 and at SIO for SR 5. Aerosol was sampled at two locations along the IB coast: from a second floor deck at the Dempsey Holder Safety Center (IBSCa, SR 1-2), at an elevation of 5 m above sea level (MASL) and 50 m from the shoreline, and at Border Field State Park (IBBFa, SR 3-4) at 20 MASL and 100 m from the shoreline (Figure 1). Aerosol was sampled at SIO near the East (landward) end of the Ellen Browning Scripps Memorial Pier at SIO (SIOPa, Figure 1). IBSCa, IBBFa, and SIOPa lie 3 km North, 2 km South, and 37 km North of the TJR mouth, respectively (Figure 1). During typical onshore winds, all aerosol sampling sites were downwind of an active surf zone with abundant wave breaking, an important source of SSA.^24^ We also observed whitecaps in the local coastal waters on multiple occasions, and they were an additional source of local SSA. Aerosol (total suspended particles) was collected in triplicate onto 47 mm quartz fiber filters in filter holders (2500 QAT-UP and 2220, Pall Corporation, New York) at 30 liters per minute for 22 h. To minimize contamination, filters were combusted at 500 °C for 2 h prior to sampling and stored in a combusted aluminum foil before deployment and after recovery. Laboratory blanks were combusted filters; field blanks were filters taken into the field, placed into filter holders, and then immediately removed. Across five rounds of sampling, we sampled coastal waters on 26 days and coastal aerosol for 21 one-day periods.

### Non-Targeted Chemical and Microbial Analyses

Aerosol and water samples were analyzed using liquid chromatography high-resolution tandem mass spectrometry (LC–MS/MS) to identify chemical species and 16S rRNA gene amplicon sequencing (16S) to identify bacteria taxa. Detailed methods are provided in the Supporting Information. LC–MS/MS was acquired as described before.^5,25^ To apply the method to aerosol samples, one 47 mm aerosol filter was extracted with 0.5 mL of methanol, followed by 2 mL of ultrapure water and sonication. Filter extracts and seawater samples were desalted *via* solid-phase extraction, followed by chromatographic separation with a C18 reversed phase column, and then two technical replicates were analyzed with a Q-Exactive quadrupole orbitrap mass spectrometer (Thermo Fisher Scientific, Bremen, Germany) in the top 5 data-dependent acquisition mode. For 16S, 1/4 of an aerosol filter or 400 μL of a water sample was extracted and sequenced using the KatharoSeq method for low biomass samples.^26^

### Data Analysis

The 16S data were processed through the QIIME 2 workflow using Qiita.^27,28^ Processing with Deblur resulted in the identification of 7627 amplicon sequencing variants (ASVs) that were annotated using GreenGenes.^29,30^ For simplicity, we refer to the ASVs as bacteria. The LC–MS/MS data were processed using MZMine2 and the Global Natural Products Social Molecular Networking (GNPS) ion identity networking workflow, producing 16822 chemical features.^31^ 2028 Level 2 compound annotations were determined from matches to mass spectral libraries, yielding an annotation rate of 0.12.^32,33^ Mass spectra were matched against the GNPS and NIS17 libraries using a minimum cosine score of 0.7 to define spectral similarity. Precursor and fragment ion mass tolerances were set to 0.01 Da, minimum matched fragment ions were set to 4, and the minimum cluster size was set to 1 (MS Cluster off). The maximum mass difference was set to 100 Da for Analogue Search. The Level 2 annotations provide tentative compound identifications, but definite confirmation requires chemical standards.

SourceTracker2 (ST2) was employed to identify potential contributions to the aerosol from the sampled waters.^34^ ST2 is a Bayesian statistical method that assigns sources to sinks on a feature-by-feature basis. ST2 was run with the aerosol samples as sinks and the water samples as sources.

In order to assess if it was possible that the air masses we sampled contained SSA from the local waters, a local particle origin for each sampling period was derived from local winds and a particle dispersion model. We used FLEXPART version 9.0, a Lagrangian particle dispersion model that simulates the release of particles into the atmosphere and uses gridded wind speeds and directions to estimate transport forward or backward in time.^35,36^ Input data were the National Centers for Environmental Protection Climate Forecast System Version 2 6 hourly products. Data were accessed from the Research Data Archive at the National Center for Atmospheric Research (https://rda.ucar.edu/datasets/ds094.0/?hash=access). The parameters selected were the FLEXPART Model Input: 1 h to 6 h forecasts including wind speeds, temperature, planetary boundary layer height, pressure, pressure reduced to MSL, and relative humidity on a 0.5 × 0.5 ° grid. We ran FLEXPART in the back trajectory mode starting at the end of each aerosol sampling period and running back to 24 h before the start of the sampling period, for a total of 46 h, sufficient to evaluate whether the sampled air mass traveled over local waters or passed over land (Figure S2). For each sampling period, 500 simulated particles were released from the sampling site at an elevation of 5 m and transported backward in time and space. Local particle origins (and aerosol samples) were classified as coming from the sea (IBa-sea; *n* = 5) or from the land (IBa-land; *n* = 5) when winds and back trajectories agreed on either; otherwise, mixed (IBa-mixed; *n* = 7) was assigned (Figure S2). A potential downside of sampling IBa from two locations on separate days is that atmospheric conditions were not identical: IBSCa sampling periods were 1 sea, 1 mixed, and 4 land; IBBFa periods were 4 sea, 6 mixed, and 1 land. Therefore, we do not compare the two IBa locations. Instead, we group the IBa data according to the local particle origin, and we target IBa-sea samples for signs of CWP in SSA. We assume that the sampled aerosol includes SSA and non-SSA during sea and mixed periods, and we refer to the sea particle populations as coastal aerosol coming from the direction of the ocean. Land periods from both IBa locations characterize continental aerosol of the region and are used for comparison. Aerosol sampling at SIO (SIOPa) had three sea periods and one mixed (Figure S2) and are used to compare against the IBa-sea and IBa-mixed samples.

### Identifying Potential Tracers of Airborne CWP

The ST2 outputs and relative abundances were used to identify potential tracers of TJRw in the sampled aerosol. Features from LC–MS/MS (chemicals) and 16S (bacteria) were ranked from IBa-sea samples, which are most likely to contain the largest SSA:non-SSA ratio. Features were not selected from IBPw because they would not necessarily be pollution-associated. In the ST2 source apportionments, for each feature in each IBa-sea sample, the SIOPw contribution was subtracted from the TJRw contribution, to prioritize features abundant in TJRw but not in SIOPw. These differences were then summed across the five IBa-sea samples to provide an initial ranking. Then, we removed features that did not meet the following criteria based on estimated, relative abundance, using MS1 peak areas for LC–MS/MS and read counts for 16S: (a) TJRw > SIOPw; (b) IBa-sea > SIOPa; and (c) IBa-sea > blank. Criterion (a) was a check on the subtraction done in the ST2 tracer ranking to identify features associated with the TJRw; criterion (b) removed features more abundant at our reference location; and criterion (c) excluded sample contaminants. Mean values were used due to the small number of samples in each of these subsets. In the 16S data, criteria (a), (b), and (c) did not remove any of the top 40 bacteria from the initial ST2 ranking. This analysis was carried out using MATLAB version 9.10.0.1602886 (R2021a).^37^

## Results and Discussion

### Sample Types Differ in Chemical and Bacterial Compositions

**Figure 2:**
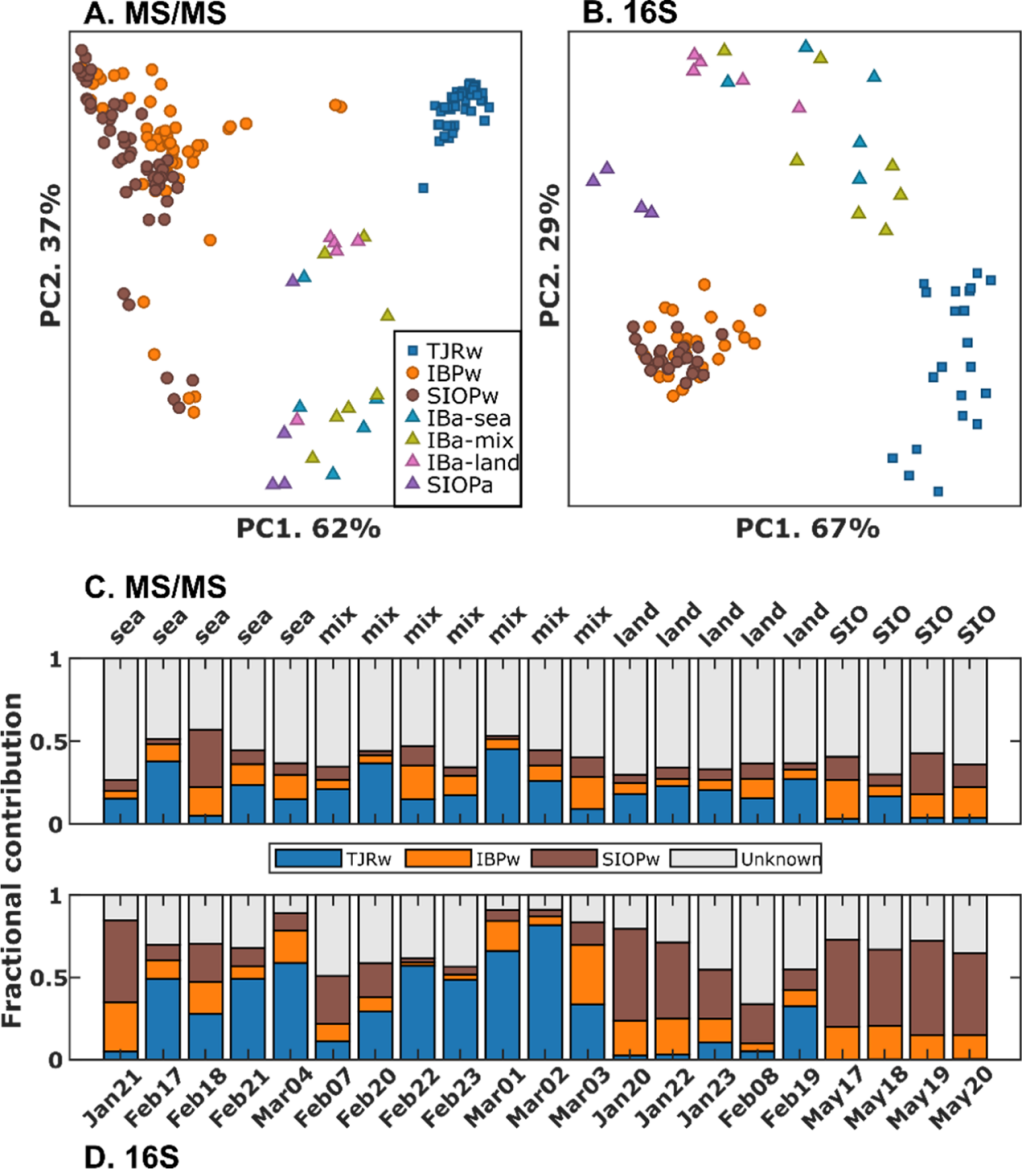
RPCA and aerosol source apportionment from the bacteria community and chemical composition. (A,B) shows RPCA (Aitchison distances) of non-targeted mass spectrometry (A) and 16S data (B). (C,D) presents ST2 results—the fractional contributions of different sources to each aerosol sample—for non-targeted mass spectrometry (C) and 16S data (D). Each bar represents one aerosol sample and is composed of the fractional contribution of molecules (C) or bacteria (D) from TJRw (blue), IBPw (orange), SIOPw (brown), and Unknown (gray) as determined by ST2. Bars align vertically between (C) and (D) and are for the same aerosol sample.

For an initial evaluation of compositional similarities and differences across the sample types, we applied Robust Aitchison principal component analysis (RPCA) to their chemical compositions and bacteria communities (Figure 2A,B). Samples that are closer together in RPCA space have more similar compositions than the samples that are further apart.^38^ In both LC–MS/MS (Figure 2A) and 16S (Figure 2B) RPCA spaces, points plot along PC2 according to the broad sample type: water or aerosol. IBPw groups with SIOPw and separates out from TJRw (Figure 2A,B), indicating that after entering IB coastal waters, TJRw did not travel North to substantially impact IBPw. PC1 appears to separate out samples according to the chemical composition (Figure 2A) or bacteria community (Figure 2B), and IBa plots closer to TJRw, whereas SIOPa plots closer to ocean water.

### IBa and TJRw Have Significant Compositional Similarities

The ST2 analysis indicates a significant overlap in the chemical species and bacteria communities found in TJRw and IBa. Figure 2C,D shows the fractional contribution of each source to each aerosol sample ST2 calculated from the chemical and bacterial compositions. According to ST2, up to 45% of the chemical composition (Figure 2C) and 82% of the bacteria community (Figure 2D) found in IBa came from TJRw, with much smaller TJRw values for SIOPa.

### Bacteria Are Effective Tracers of Airborne CWP

**Figure 3:**
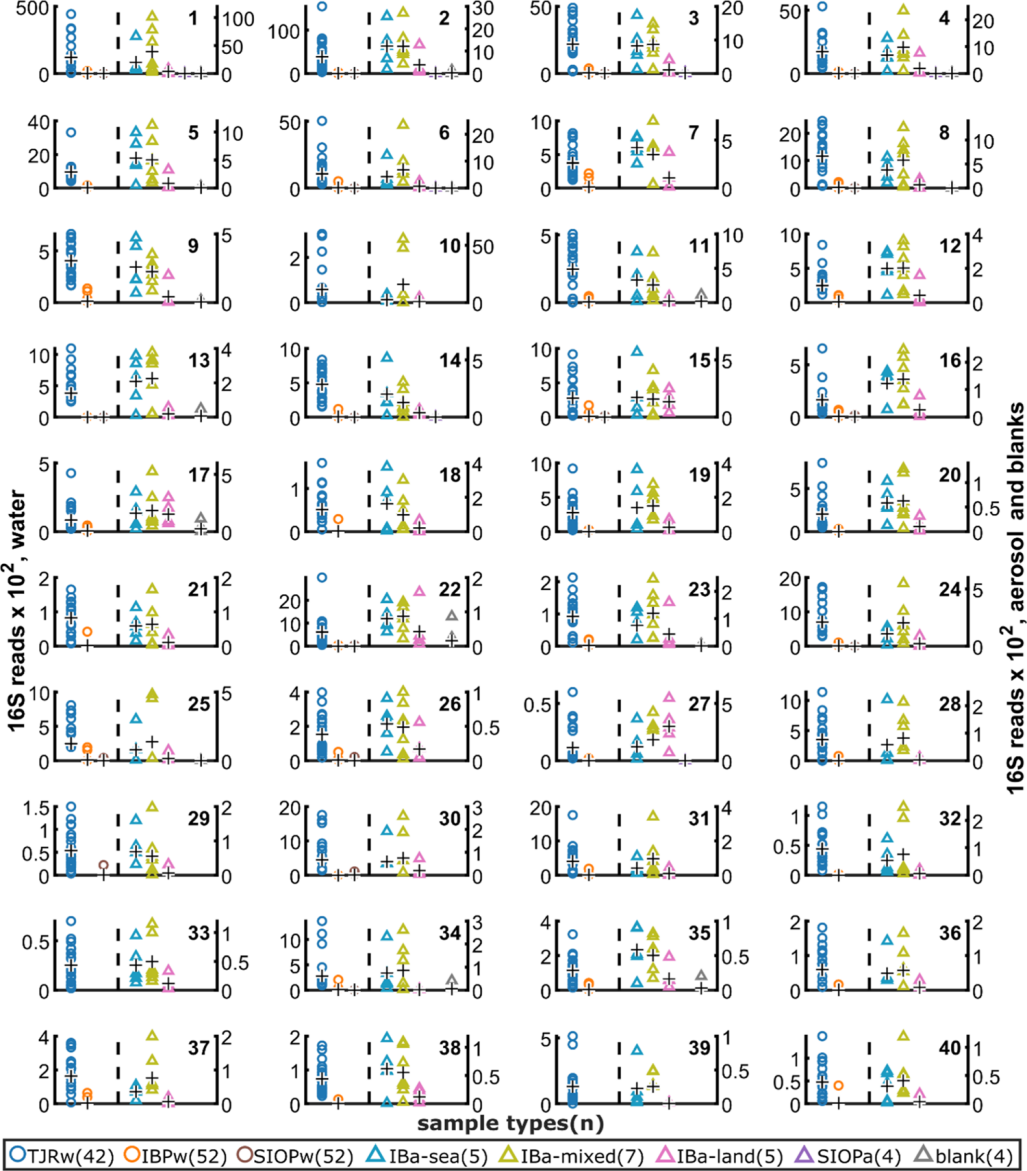
Relative abundance across sample types for the 40 potential tracer bacteria of the polluted TJR in IB aerosol. Each subplot represents a single bacterium (ASV). Each point is the read count of the bacterium in one sample. Sample types (and # of samples) are provided in the legend. Water samples plot on the left axes; aerosol samples and blanks plot on the right axes. Each black “+” denotes the sample type mean. For each bacterium/subplot, we look for the following tracer pattern: [TJRw] > [SIOPw] and [IBa-sea] > [IBa-land, SIOPa, and blanks]. Most bacteria here show a tracer pattern.

**Figure 4:**
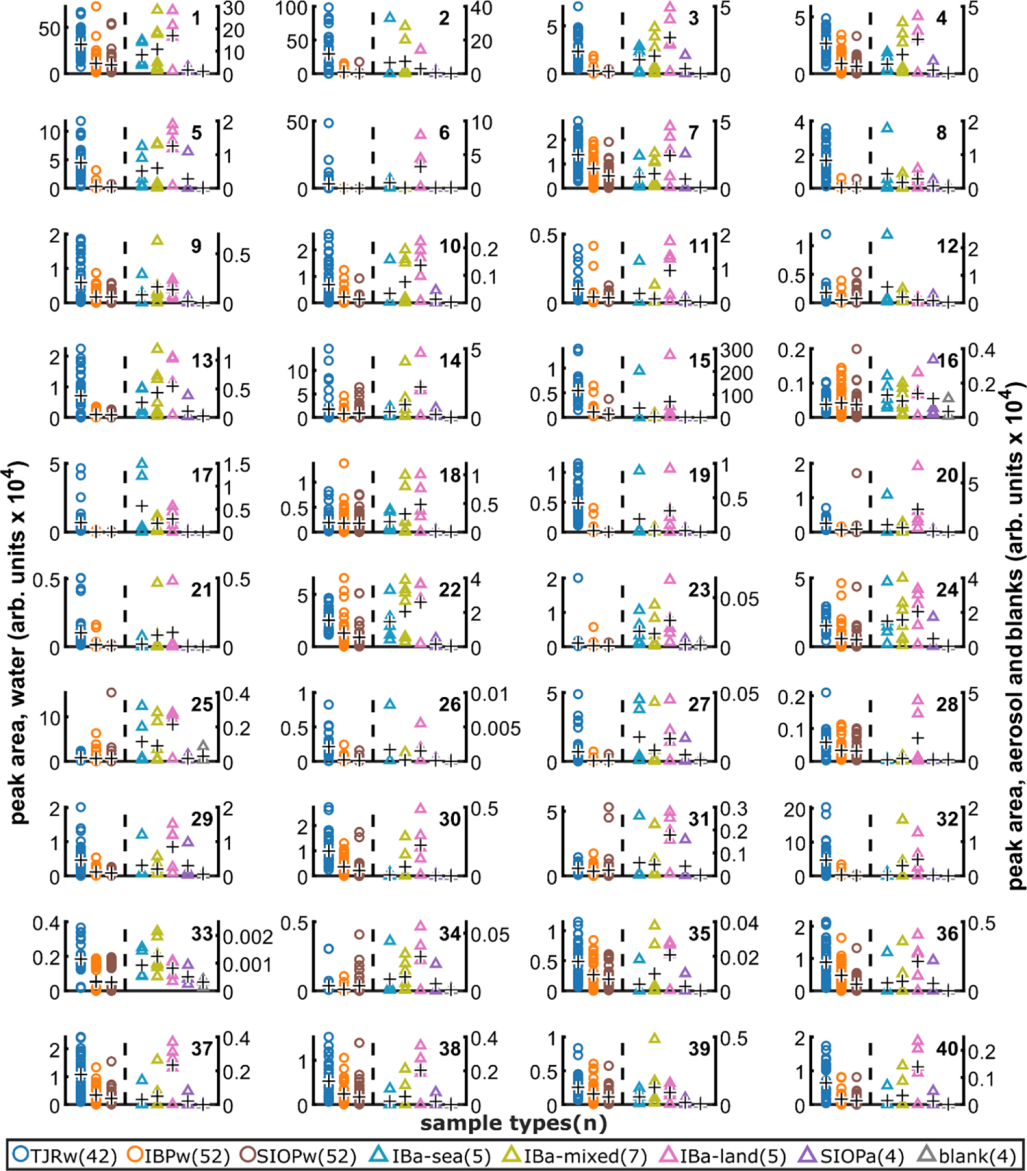
Relative abundance across sample types for the 40 chemical links between the polluted TJR and IB aerosol. Each subplot represents a single compound. Each point is the MS1 peak area of the compound in a sample. Sample types (and # of samples) are provided in the legend. Each black “+” denotes the sample type mean. Water samples plot on the left axes; aerosol samples and blanks plot on the right axes. Most compounds lack a tracer pattern of [TJRw] > [SIOPw] and [IBa-sea] > [IBa-land, SIOPa, and blanks] due to high IBa-land relative abundance. This implies that they have multiple sources, so we do not consider them as tracers but as chemical links between TJRw and IBa.

Evaluation of the top 40 bacteria identified by our tracer selection criteria supports that most are effective tracers of airborne CWP. Although amplicon sequencing is not appropriate for absolute quantitation, we used 16S read counts to estimate and compare the relative abundance of each tracer bacterium across the different sample types, with a focus on the local particle origin (Figure 3). In each feature, we look for a tracer pattern: TJRw > SIOPw, IBa-sea > IBa-land, SIOPa, and the blanks (Figures 3 and 4). Although in a few cases IBa-sea < IBa-land (# 27) or IBa-sea ≈ IBa-land (#s 10, 15, 17), in general, these 40 bacteria show strongest associations with TJRw and IBa-sea and we consider them tracers of the polluted TJRw in IBa (Figure 3). We present these data as direct observation of bacteria in the polluted TJR flowing into coastal waters, transferring to the atmosphere in SSA, and returning in onshore winds.

### Tracer Bacteria Taxonomies Link Them to Sewage

Although mere library matches to the GreenGenes database, the taxonomic identities of the tracer bacteria link 26 of them to sewage (Table S1).^29^ These taxa include bacteria attributed specifically to TJ sewage and sewage foam and taxa containing pathogenic and antimicrobial-resistant members (Table S1 and references within). The genus *Arcobacter* appears eight times in the list and contains members that are pathogens, resistant to antimicrobials, and/or commonly found in sewage and sewage-contaminated waters.^41−43^*Acinetobacter* spp. are found in hospital infections and sewage and are increasingly resistant to antibiotics.^44,45^ Fifteen of the 40 tracer bacteria are nonfermenting Gram-negative bacilli, a group of bacteria that contains many pathogens.^46^ The *Bacteroides* genus contains the most common gene marker for human fecal pollution, HF183.^47,48^*Acinetobacter* spp. and *Alkanindiges* spp. combinedly appear seven times in the list and are dominant in biofoam at wastewater treatment plants.^49^ Their hydrophobic cell surfaces may cause their enrichment in foam and preferential aerosolization, as previously observed for *Actinobacteria* in SSA.^50,51^

### Tracer Bacteria Independently Linked to TJR and TJ Sewage

The bacteria communities of the TJR and a sewage outfall South of TJ were characterized with 16S amplicon sequencing in the same year we sampled.^12^ The most abundant taxa were *Acidovorax*, *Bacteroides*, *Cloacibacterium*, *Comamonas*, *Macellibacteroides*, and the potentially pathogenic genera *Acinetobacter*, *Aeromonas*, and *Arcobacter*. Nineteen of our 40 tracer bacteria match these taxa *via* their GreenGenes assignments (Table S1), further supporting that these bacteria are effective tracers of TJ sewage aerosolized in SSA.

### Chemical Links between CWP and IB Aerosol in Onshore Winds

Applying the same feature ranking criteria to the LC–MS/MS data identified chemical links between CWP and IB aerosol in onshore winds. As done for the tracer bacteria, we evaluated the selected LC–MS/MS features by comparing relative abundance, from MS1 peak areas, for each feature across the different sample types, and looking for compounds that meet our tracer criteria: TJRw > SIOPw and IBa-sea > IBa-land, SIOPa, and blanks (Figure 4). We report the top 40 chemical species with annotations (Level 2), excluding likely misannotations (*n* = 2), compounds that did not return clear search results (*n* = 8), and polyethylene glycols, because they are common sample contaminants (Table S2). Although some compounds show a weak tracer signal (#s 2, 8, 12, and 17), most compounds show IBa-sea ≈ SIOPa and IBa-sea < IBa-land (Figure 4). This implies that these compounds in IBa have marine and continental sources, and therefore, we do not consider them explicit tracers of TJRw pollution in SSA. However, they are chemical links shared between CWP and coastal aerosol in onshore winds, and we use them to provide chemical information on the same aerosol populations that contained our tracer bacteria.

### Anthropogenic Compounds Dominate Chemical Links

Tentative Level 2 annotations for most of the selected chemical links are anthropogenic compounds, indicating a polluted aerosol population (Table S2). Industrial chemicals are common in the list, including flame retardants, paints, solvents, plasticizers, cleaning products, and personal care products, as well as known irritants and common environmental pollutants. These compounds may have reached the TJR by direct discharge from industrial facilities or from urban-industrial stormwater runoff. Other chemical species are human-associated and indicative of sewage, such as caffeine and vitamin K2. Monopalmitolein (9c), lumichrome, and 5(*Z*),8(*Z*),11(*Z*)-eicosatrienoic acid methyl ester are marine-associated, indicative of SSA. We annotated 160 drugs, 21 drug metabolites, 179 food compounds, 15 food additives, 36 biocides, 487 natural products, and 6 compounds from personal care products in our LC–MS/MS dataset as Level 2 IDs (*n* = 497; list provided at https://doi.org/10.6075/J07944V3) and tested them as tracers (Figure S6).^32^ Aerosols from the land appear to be the dominant source for all of them, and drugs have previously been detected in urban aerosol.^53,54^

### Evaluating Bacteria versus Chemicals as Tracers

This study found bacteria to be more effective than chemicals at identifying signs of TJRw aerosolizing in SSA. The chemical and bacterial ST2 source apportionments significantly differed (Figure 2C,D), likely because bacteria are only found in larger aerosols, whereas chemical compounds are present in all aerosols.^39,40^ Larger aerosols have shorter residence times in the atmosphere, so the airborne bacteria community is strongly influenced by local sources. In our tracer evaluations, the chemicals are more ubiquitous across the sample types compared to the bacteria (Figures 3 and 4). IBa-land samples yielded much more total LC–MS/MS signal than IBa-sea and IBa-mixed samples (Figure S3A), limiting the utility of chemicals as tracers of TJRw in IBa. Greater molecular diversity and abundance in polluted/continental aerosol versus non-polluted/marine aerosol have been previously observed.^52^ Normalization of microbiome and mass spectrometry data are often used to correct for variations in total signal strength across samples.^70,71^ Here, normalizing to total 16S read counts or LC–MS/MS peak area per sample (Figures S4 and S5) yielded more tracer signatures in the LC–MS/MS data, but we feel that the usage of non-normalized data is more appropriate for this study in order to include differences in the contributions of marine versus continental aerosol to IBa (Figure S3). In comparison, 16S read counts were similar across the IBa samples (Figure S3B), and cell counts, a better measure of bacteria amount, were higher in IBa-sea and IBa-mixed versus IBa-land (Figure S7), suggesting that bacteria are particularly useful as tracers of SSA.

### Our Results in Context

Our findings are in agreement with the ability of SSA to transfer diverse chemical compounds and microorganisms from the ocean to the atmosphere, including naturally occurring toxins, like brevetoxin from red tides, and artificial toxins, like perfluoroalkyl acids.^19,55−58^ Aerosolization of sewage by aeration and bubble bursting has been observed at wastewater treatment plants, open wastewater canals, spray irrigation, and the aeration of polluted waters but not by SSA.^59−65^ Direct aerosolization from the TJR may occur, but it is likely to be a much smaller source than SSA from the surf zone, a significant aerosol source, and from whitecaps in local waters.^24^ TJR aerosol would have been downwind during IBa-sea samples but could have been a minor contribution to IBa-mixed and IBa-land samples. The sequencing of Central California coastal aerosol and SSA isolated in the laboratory-identified taxa contain pathogenic strains but of unknown origin.^51,66^ We build on this work by linking bacteria and compounds in coastal aerosol to a major CWP source.

### Significant Contributions of Airborne Bacteria

**Figure 5:**
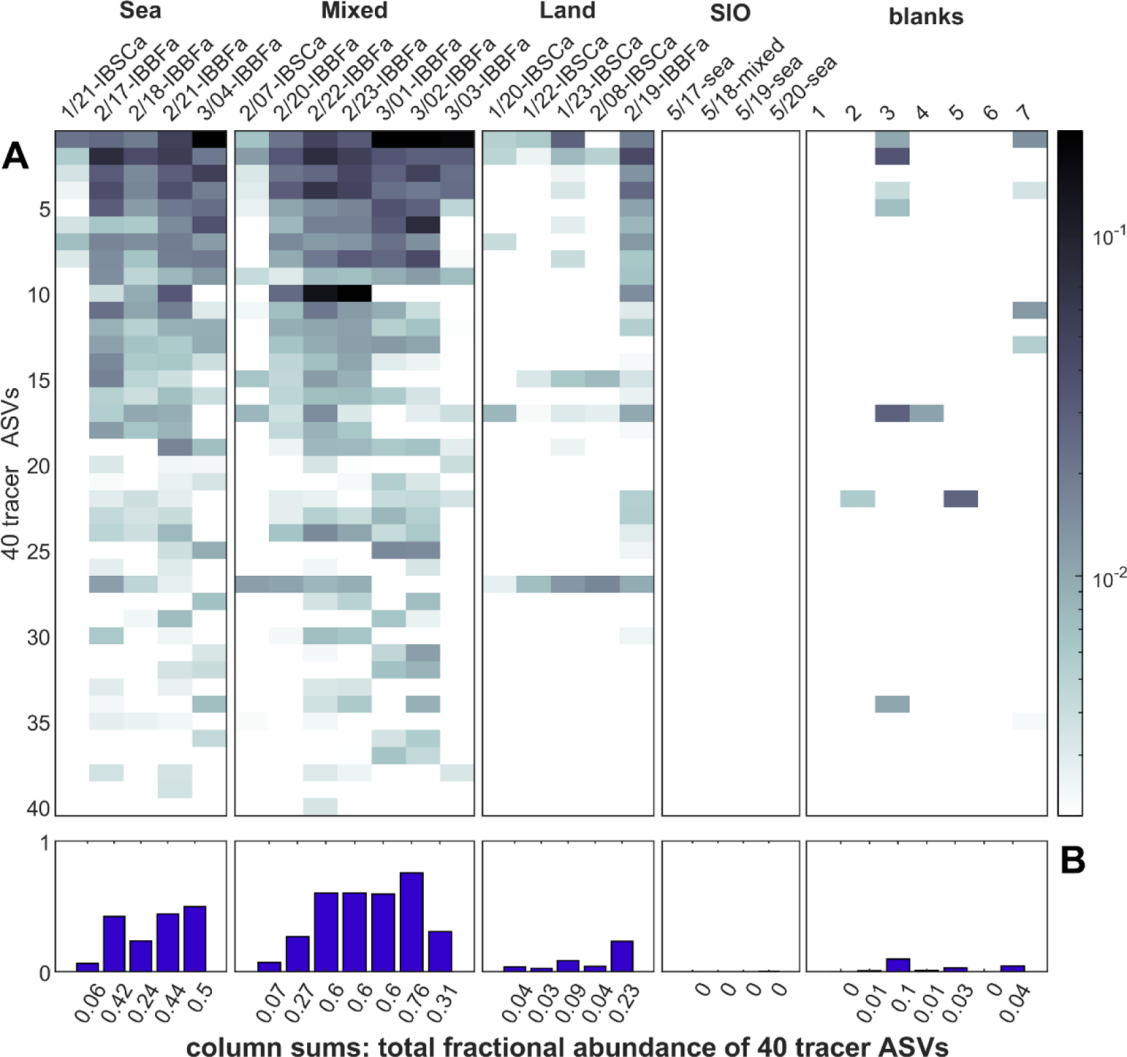
Individual and combined fractional abundance of the 40 tracer bacteria grouped by the local particle origin. (A) Heatmap of individual fractional abundances, with bacteria (ASVs) in rows and samples as columns. Fractional abundance was calculated by dividing the read count for each ASV in each sample by the total reads for that sample. (B) Sums of columns in (A), giving the combined fractional contribution of the 40 bacteria to the entire sample.

To investigate the magnitude of CWP’s contribution to coastal aerosol, we examined the individual and combined fractional abundance of our 40 selected bacteria in the aerosol samples (Figure 5). Like Figure 3, the tracer bacteria are most abundant in IBa-sea and IBa-mixed samples, most of which were collected at IBBFa. These bacteria are highest in IBa-mixed periods possibly because they encountered the most ideal conditions for the transfer of CWP in SSA and collection, despite mixed winds: the highest bacterial pollution levels in the upwind waters and greatest SSA production. IBBFa shows higher levels than IBSCa, suggesting that IBBFa was better located, possibly due to Northwest winds and IBBFa lying South of the TJR mouth (Figures 1, S1, and S2). Together these 40 bacteria comprise 41% on average and up to 76% of the 16S reads in the 12 total IBa-sea and IBa-mixed samples (Figure 5B). This demonstrates that a significant fraction of the airborne bacteria breathed by coastal communities can come from CWP, and this should be considered for public health along coastlines.

### Implications

This study presents evidence of CWP transferring to the atmosphere in SSA and calls attention to potential public health impacts that need to be further explored. The multiple environmental conditions that transport pollution through this exposure pathway are under investigation.^67,68^ Future studies will focus on more comprehensive sampling and target specific chemicals and pathogens. This environmental and public health problem is expected to grow as our changing climate brings more extreme precipitation and CWP events.^15^ This work provides further justification for improving and monitoring coastal water and air quality along the TJ–SD coastline and other populated coastlines worldwide.

## Supporting Information Available

Tracer bacteria of the polluted TJR in IB aerosols coming from the sea, chemical links between the TJR and IB aerosols coming from the sea, environmental conditions, local particle origins from local winds and FLEXPART back trajectories, relative signal strength across sample types, relative normalized abundance across sample types for the 40 potential tracer bacteria of the polluted TJR in IB aerosol, relative normalized abundance across sample types for the 40 chemical links between the polluted TJR and IB aerosol, relative abundance by sample type for annotated compound classes, cell counts of heterotrophic bacteria abundance in water and air, data availability, and supplemental methods (PDF)
