# Supplementary material for: Bacterial and Chemical Evidence of Coastal Water Pollution from the Tijuana River in Sea Spray Aerosol
Source: Environ Sci Technol. 2023 Mar 2;57(10):4071–81. doi: 10.1021/acs.est.2c02312 (PMC10018732; doi:10.1021/acs.est.2c02312)
Supplement: Supplementary file 1 — es2c02312_si_001.pdf [file es2c02312_si_001.pdf]

Supporting Information for:

Bacterial and chemical evidence of coastal water pollution from the Tijuana River in sea spray aerosol

Matthew A. Pendergraft,[1] Pedro Beldá-Ferre[2], Daniel Petras[1,3,4], Clare K. Morris[1,5], Brock A. Mitts[5], Allegra T. Aron[3], MacKenzie Bryant[2], Tara Schwartz[2], Gail Ackermann[2], Greg Humphrey[2], Ethan Kaandorp[6], Pieter C. Dorrestein[2,3,7], Rob Knight[2,7,8,9], Kimberly A. Prather[1,5]**

1. Scripps Institution of Oceanography, University of California, San Diego, La Jolla, CA 92037, USA

2. Department of Pediatrics, University of California, San Diego, La Jolla, CA 92093, USA

3. Collaborative Mass Spectrometry Innovation Center, Skaggs School of Pharmacy and Pharmaceutical Science, University of California, San Diego, La Jolla, CA 92093, USA

4. CMFI Cluster of Excellence, Interfaculty Institute of Microbiology and Medicine, University of Tuebingen, Tuebingen, 72076, Germany

5. Department of Chemistry and Biochemistry, University of California, San Diego, CA 92093, USA

6. independent researcher

7. Center for Microbiome Innovation, University of California, San Diego, La Jolla, CA 92093, USA

8. Department of Bioengineering, University of California, San Diego, La Jolla, CA 92093, USA

9. Department of Computer Sciences and Engineering, University of California, San Diego, La Jolla, CA 92093, USA

Corresponding Authors

*Kimberly A. Prather ; kprather@ucsd.edu

*Matthew A. Pendergraft ; mpenderg@ucsd.edu

CONTENTS

Number of pages: 34

Number of figures: 7
Number of tables: 2
Data Availability
Supplemental Methods
References for the Supporting Information

Key Words
water pollution, coastal, sea spray aerosol, pathogen, airborne exposure, 16S, mass spectrometry, Imperial Beach, Tijuana, Tijuana River, Scripps Institution of Oceanography

Synopsis

Here we use bacteria from 16S amplicon sequencing and chemicals from tandem mass spectrometry to track the polluted Tijuana River flowing into coastal waters and transfering to the atmosphere in sea spray aerosol.

TABLES S1-S2

Table S1. Tracer bacteria of the polluted Tijuana River in IB aerosols coming from the sea.

#	Taxonomy	Sewage	pathogens	Other	Sources
**1**	g_Arcobacter s_cryaerophilus	Yes, TJ	Yes	foodborne, antimicrobial resistance	1–4
**2**	g_Acinetobacter	Yes, dominant, TJ, foam	Yes	NFGNB	4,5
**3**	g_Arcobacter	Yes, dominant, TJ	Yes	foodborne, antimicrobial resistance	1–4
**4**	f_Aeromonadaceae	Yes, TJ	Yes	fresh & brackish water	6
**5**	g_Acidovorax	Yes, TJ	Yes	NFGNB	7
**6**	g_Arcobacter s_cryaerophilus	Yes, TJ	Yes	foodborne, antimicrobial resistance	1–4
**7**	g_Flavobacterium	No	No	ubiquitous; IB seawater	1,8
**8**	g_Arcobacter	Yes, dominant, TJ	Yes	foodborne, antimicrobial resistance	1–4
**9**	g_Comamonas	Yes, TJ	rarely	ubiquitous; NFGNB	1,4
**10**	g_Flectobacillus	No	No	freshwater , microplastic biofilms	9,10
**11**	g_Pseudomonas	ubiquitous	rarely	NFGNB	11
**12**	f_Pseudomonadaceae	ubiquitous	rarely	NFGNB	11
**13**	g_Hydrogenophaga	Yes, strongly	No	hydrogen-oxidizing aerobe	12–15
**14**	g_Aquabacterium	Yes	No	common to municipal water	16
**15**	g_Acinetobacter s_lwoffii	Yes, dominant, TJ, foam	Yes	NFGNB, antimicrobial resistance	4,16–18
**16**	g_Shewanella	ubiquitous	rarely	NFGNB	19,20
**17**	o_Clostridiales	Yes	Yes	common in gut	4,21–23
**18**	f_Pseudomonadaceae	ubiquitous	rarely	NFGNB	24
**19**	o_Fusobacteriales	No	Yes	humans, marine, terrestrial, infections	25–28
**20**	g_Bacteroides	Yes, TJ	Yes	fecal indicator, antimicrobial resistance	1,29–31
**21**	g_Rheinheimera	No	No	marine water & sediment; freshwater; soils	32
**22**	g_Acinetobacter s_johnsonii	Yes, dominant, TJ, foam	No	NFGNB , antimicrobial resistance	18
**23**	f_Pseudomonadaceae	ubiquitous	rarely	NFGNB	24
**24**	g_Acinetobacter	Yes, dominant, TJ, foam	Yes	NFGNB	4,5
**25**	g_Hydrogenophaga	Yes, strongly	No	hydrogen-oxidizing aerobe	12–15
**26**	g_Arcobacter	Yes, dominant, TJ	Yes	foodborne, antimicrobial resistance	1–4
**27**	g_Alkanindiges	Yes, foam	No	freshwater, terrestrial	33–35
**28**	g_Arcobacter	Yes, dominant, TJ	Yes	foodborne, antimicrobial resistance	1–4
**29**	f_Rhodocyclaceae g_C39	No	No	freshwater	36,37
**30**	g_Acinetobacter s_lwoffii	Yes, dominant, TJ, foam	Yes	NFGNB, antimicrobial resistance	4,17,18
**31**	g_Arcobacter	Yes, dominant, TJ	Yes	foodborne, antimicrobial resistance	1–4
**32**	f_Pseudomonadaceae	ubiquitous	rarely	NFGNB	24
**33**	g_Aeromonas s_sharmana	No	No	freshwater	38
**34**	g_Arcobacter	Yes, dominant, TJ	Yes	foodborne, antimicrobial resistance	1–4
**35**	f_Pseudomonadaceae	ubiquitous	rarely	NFGNB	24
**36**	g_Zoogloea	Yes	No	dominant in sewage at lower temps.	39
**37**	g_Acinetobacter	Yes, dominant, TJ, foam	Yes	NFGNB	4,5
**38**	f_Aeromonadaceae	Yes, TJ	Yes	fresh & brackish water	6
**39**	f_Comamonadaceae	some genii Yes, some No	rarely	aquatic, soil, natural & industrial environs	40
**40**	g_Paludibacter	Yes	No	rice field, sewage	41,42

Notes: Sewage – sewage associated. Pathogens – taxon contains human pathogens. Foam – associated with sewage/wastewater foam. NFGNB – nonfermenting gram-negative bacilli. TJ – associated with Tijuana sewage. Sources are provided in the Supporting Information.

Table S2. Chemical links between the Tijuana River and IB aerosols coming from the sea.

#	Annotation	Tags	Sources
**1**	Tris(2-butoxyethyl) phosphate	irritant; pollutant; flame retardant; polishes/waxes; water treatment	43,44
**2**	N-cyclohexylcyclohexanamine	irritant; used in paints, varnishes, detergents; xenobiotic metabolite	44
**3**	Tributyl phosphate	irritant; solvent; flame retardant; plasticizer; antifoaming agent	45,46
**4**	1-Oleoyl-2-acetyl-sn-glycerol	reagent chemical	47
**5**	Galaxolidone	fragrance metabolite; sewage associated	48,49
**6**	Lauramine oxide	industrial chemical (zwitterion surfactant)	50
**7**	2-Linoleoyl glycerol	natural product	44
**8**	2,4,7,9-Tetramethyl-5-decyne-4,7-diol (Surynol 104)	industrial surfactant; defoaming; adhesives; water-based coatings	51
**9**	Aleuritic acid	shellac; perfume; personal care	52
**10**	S-Hydroprene	insecticide	53
**11**	Conjugated linoleic acid (10E,12Z)	general metabolite; dietary supplement	53,54
**12**	Prostaglandin F2alpha-1,15 lactone	eicosanoid; hormone mimic; found in mammals	55
**13**	Acetyl tributyl citrate	plasticizer in drugs, food wrap, cosmetics, medical tubes, toys	56
**14**	Myristamine oxide	hair conditioner; soap; cleaners	44
**15**	Triphenylphosphine oxide	industrial chemical; catalyst	57
**16**	Monopalmitolein (9c)	cyanobacteria	72
**17**	Vitamin K2	essential vitamin; in bacteria, fermented foods, meat, dairy, eggs	58
**18**	Fesoterodine fumarate	drug	44
**19**	8-Acetyl-7-methoxycoumarin	reagent drug	44,59
**20**	11beta-Prostaglandin E2	common in mammalian tissue	60,61
**21**	Desbenzyldonepezil	drug metabolite	62
**22**	Butaprost free acid	reagent chemical	63
**23**	Di(3,7-dimethyl-1-octyl) phthalate	plasticizer, solvent	64
**24**	4alpha-Hydroxystanozolol	drug metabolite	65
**25**	N-Lauroylsarcosine	cleaning products, hair conditioner	44
**26**	17-Phenyltrinorprostaglandin A2	synthetic prostaglandin analog; reagent	66
**27**	Sorbitane monostearate	in-source fragment of Polysorbate 60, a food & drug emulsifier	44
**28**	Dibenzylamine	petroleum lubricants; synthetic rubber manufacturing	44
**29**	5(Z),8(Z),11(Z)-Eicosatrienoic acid methyl ester	algae	67
**30**	Biotin	general metabolite	68
**31**	3,5-bis(1,1-dimethylethyl)-4-hydroxy-Benzoic acid	flame retardants; adhesives; cables	44
**32**	Caffeine	human associated	44
**33**	Dibutyl phthalate	plasticizer; environmental contaminant; teratogen; metabolite	44
**34**	Adenosine	naturally occurring in humans, also a drug, metabolite	44
**35**	Lumichrome	marine; plant metabolite	44,69
**36**	Warfarin	drug; pesticide	44
**37**	17alpha-Dihydroequilin 3-sulfate	drug	70
**38**	(-)-Riboflavin	essential human nutrient	44
**39**	13,14-dihydro-15-keto-PGF1	human metabolite	71
**40**	Methyldopa	drug	44

Notes: Sources are provided in the Supporting Information.

FIGURES S1-S7

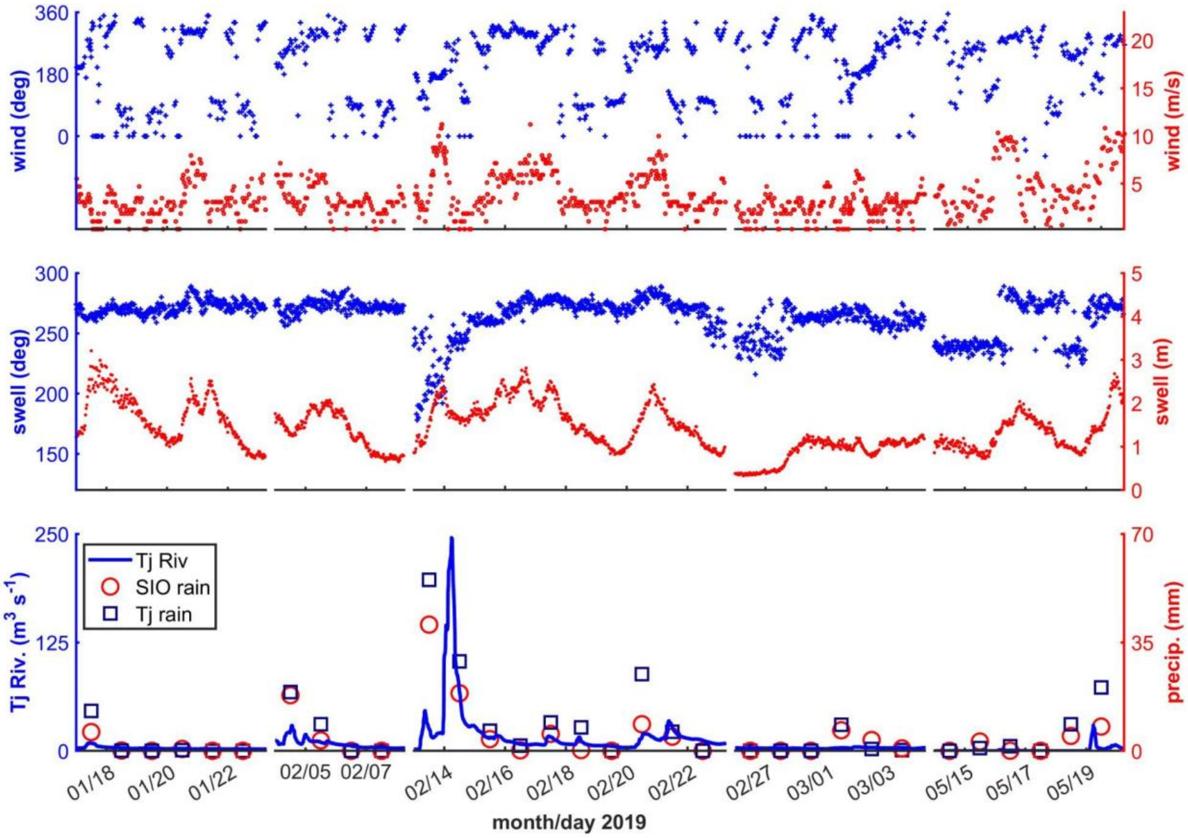

Figure S1. Environmental conditions. Sampling periods and aerosol sampling locations were: Jan. 19-23 IBSCa, Feb. 6-8 IBSCa, Feb. 16-23 IBBFa, Feb. 28 - Mar. 4 IBBFa, May 16-20 SIOPa. Aerosol sampling periods in Imperial Beach followed rain events to target coastal water pollution. The SIOPa aerosol sampling period in La Jolla included some light rain.

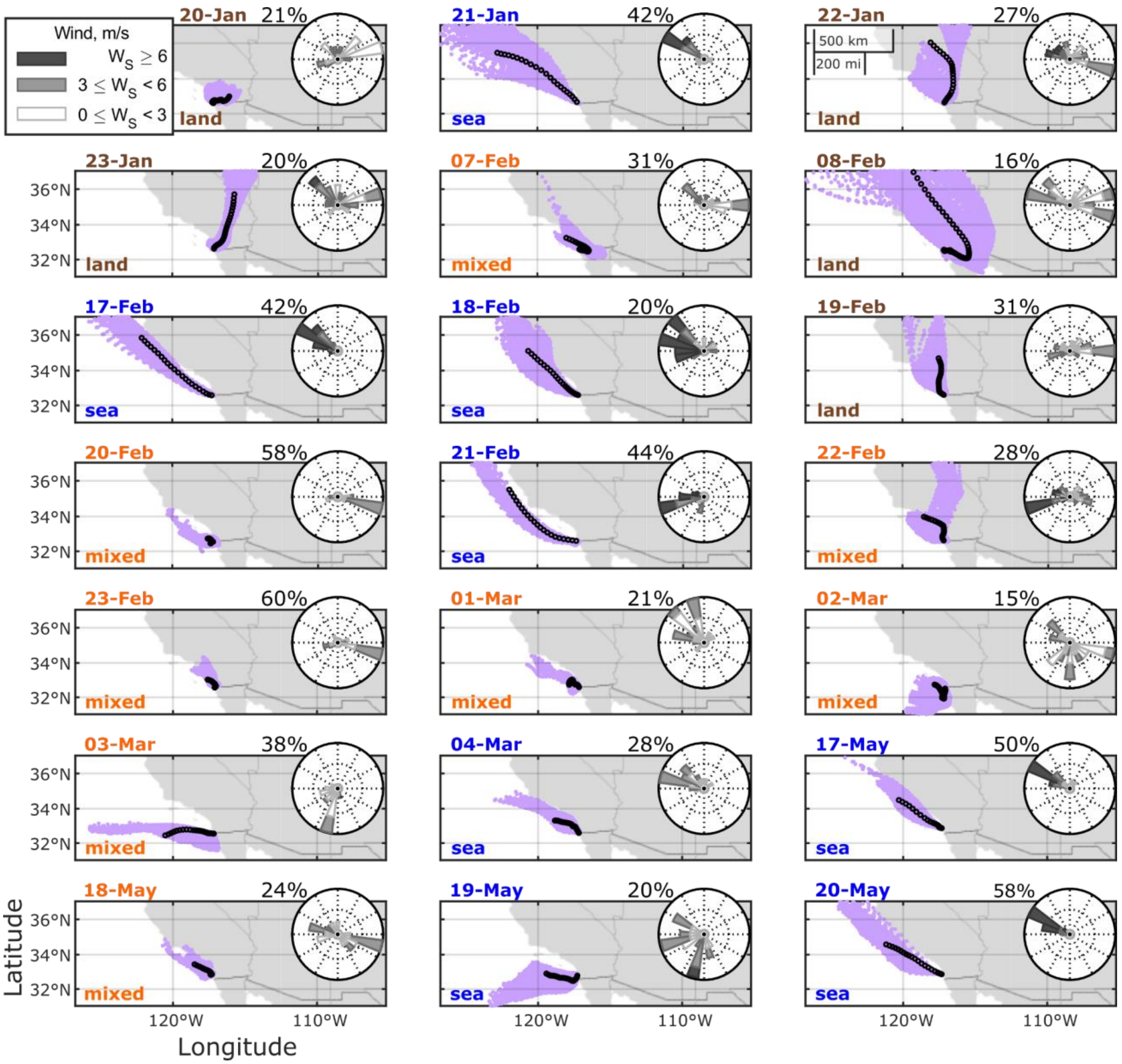

Figure S2. Local particle origins from local winds and FLEXPART back trajectories. Winds are from each sampling period, from a local meteorological station. FLEXPART back trajectories are from 24 hs prior to the initiation of each aerosol sampling period through to the end of the 22 hs sampling period. When both local winds and back trajectories agree on a land or sea origin, the corresponding classification was given. Otherwise, "mixed" was assigned. This is to characterize the origin of locally produced aerosols.

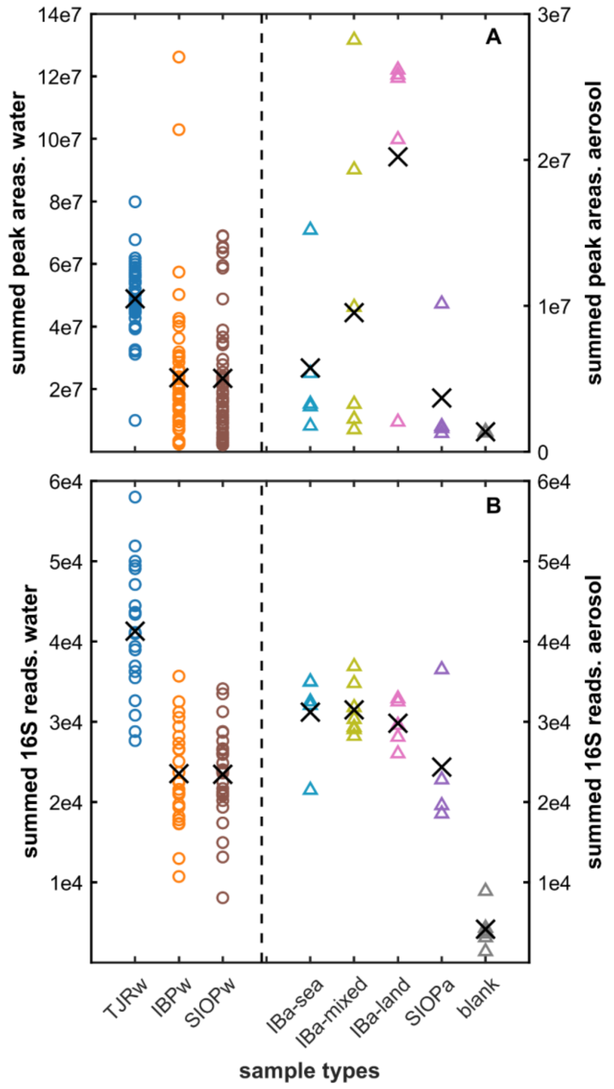

Figure S3. Relative signal strength across sample type. The total number of MS/MS peak areas (A, top) and 16S reads (B, bottom) were summed for each sample. Water samples, grouped by sample type, are on the left of the dotted line and aerosol samples, grouped by local particle origin and location, and blanks are on the right of the dotted line. X's denote means. Water and aerosol plot on separate y-axes in (A). Note the data plotted here are already blank corrected.

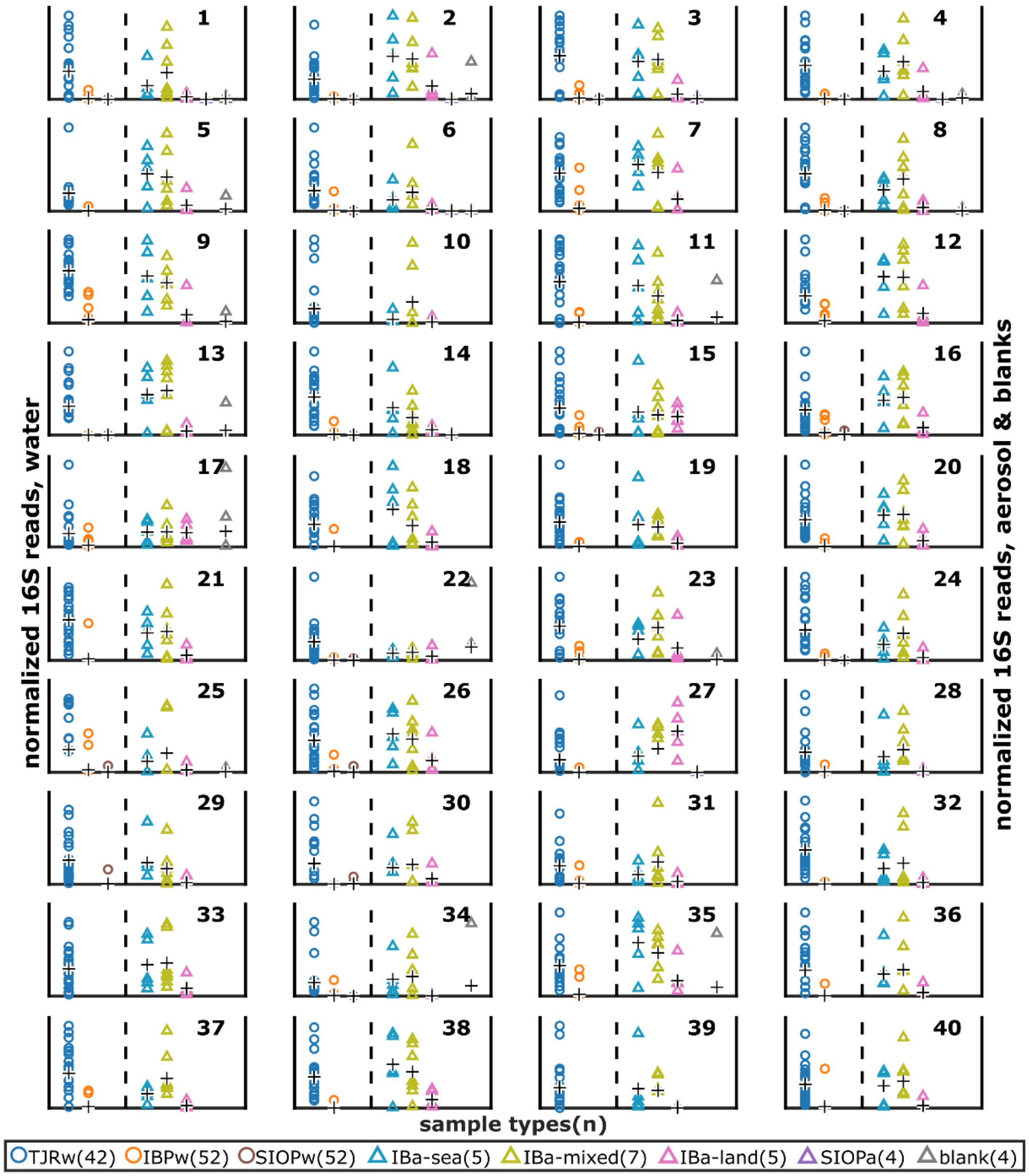

Figure S4. Relative normalized abundance across sample types for the 40 potential tracer bacteria of the polluted Tijuana River in IB aerosol. Each subplot represents a single bacterium (ASV). Each point is the read count of the bacterium in one sample, divided by the total reads of the sample. Sample types (and # of samples) are provided in the legend. Each black cross (+) denotes the sample type mean. Water samples plot on the left axes; aerosol samples and blanks plot on the right axes. For each bacterium/subplot, we look for the following tracer pattern: [TJRw] > [SIOPw] and [IBa-sea] > [IBa-land, SIOPa, and blanks].

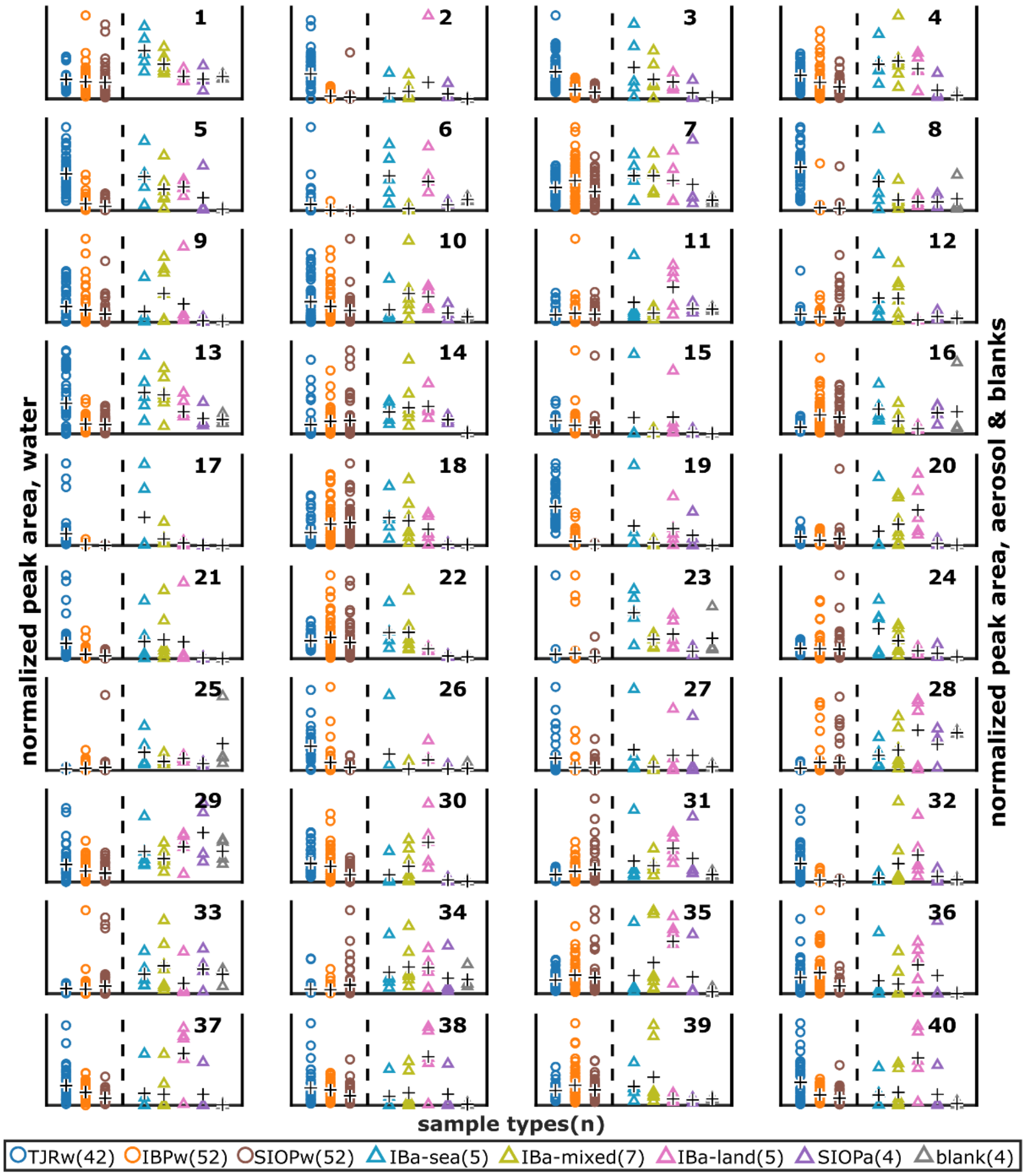

Figure S5. Relative normalized abundance across sample types for the 40 chemical links between the polluted Tijuana River and IB aerosol. Each subplot represents a single compound. Each point is the MS1 peak area of the compound in a sample divided by total MS1 peak areas for the sample. Sample types (and # of samples) are provided in the legend. Each black cross (+) denotes the sample type mean. Water samples plot on the left axes; aerosol samples and blanks plot on the right axes. Most compounds lack a tracer pattern of [TJRw] > [SIOPw] and [IBa-sea] > [IBa-land, SIOPa, & blanks] due to high IBa-land relative abundance. This implies they have

multiple sources so we do not consider them as tracers but as chemical links between TJRW and IBa.

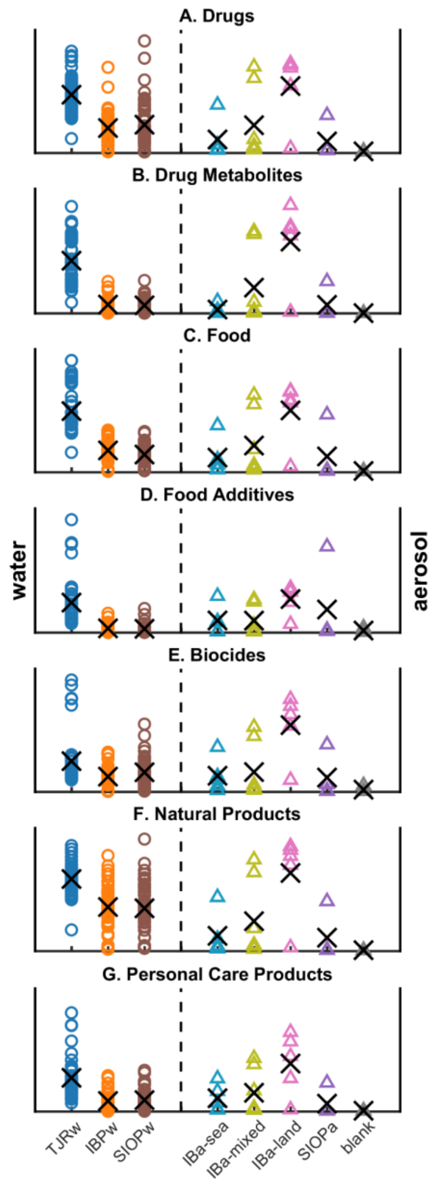

Figure S6. Relative abundance by sample type for annotated compound classes. We annotated 160 drugs, 21 drug metabolites, 179 food compounds, 15 food additives, 36 biocides, 487 natural products, and 6 compounds from personal care products in our MS/MS dataset as level 2 IDs (LIT; n=497). For each sample, we summed the peak area for all compounds in each group, and

present them separated by sample type. Water samples are on the left of the dashed line and plot on the left axis. Aerosols and blanks are on the right of the dashed line and plot on the right axis. Black X's denote mean values.

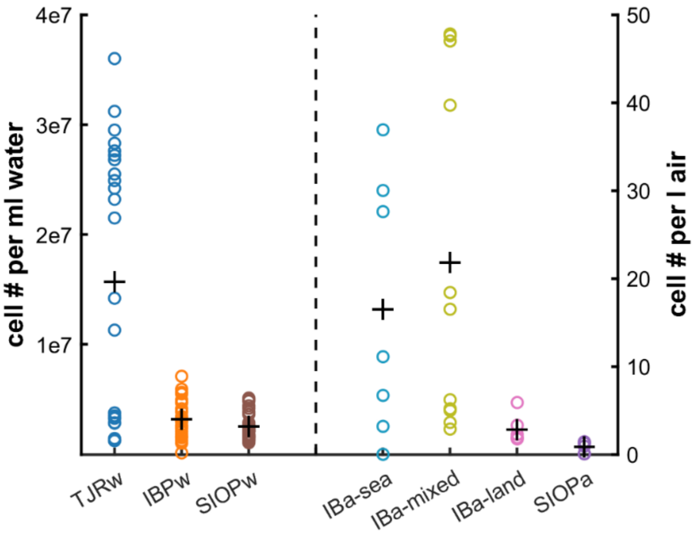

Figure S7. Cell counts of heterotrophic bacteria abundance in water and air. Aerosol samples were collected into liquid using the Series 100 Universal Spot Sampler (model SS110A; Aerosol Devices Inc, Fort Collins, CO). Cell counts were achieved using flow cytometry [92]. Black crosses are means for each sample type.

Data Availability

All MS/MS data can be found on the Mass spectrometry Interactive Virtual Environment (MassIVE) at https://massive.ucsd.edu/ with the identifier MSV000083889. Molecular Networking and Spectrum Library Matching results can be found online at GNPS under the following links:

https://gnps.ucsd.edu/ProteoSAFe/result.jsp?task=fd75a1a473864a7ab6deb29875a9a82c&view=advanced_view

https://gnps.ucsd.edu/ProteoSAFe/status.jsp?task=2edebb51fbc34f8db8f5572666e4dde2

All 16S rRNA amplicon sequencing data and sample metadata are archived and available at the European Nucleotide Archive European Molecular Biology Laboratory European Bioinformatics Institute (EMBL-EBI) under the primary accession number of PRJEB46560 and secondary accession number of ERP130752: https://www.ebi.ac.uk/ena/browser/view/PRJEB46560 and also on QIITA under project ID 12758 [72]

Other data and code are in the University of California San Diego Library's Digital Collections and are available at https://doi.org/10.6075/J07944V3

Supplemental Methods

Water Sampling

Water sampling occurred daily using an acid cleaned 5 gal high density polyethylene bucket (Home Depot, Atlanta, GA) with a rope attached to the handle. The bucket and rope were rinsed between sampling sites and thoroughly cleaned at the end of each day with solvent and scid. A separate bucket was used for sampling the heavily polluted Tijuana River. At each site a bucket was lowered into the water, raised, dumped to rinse, then lowered again to collect the sample. Coastal seawater was sampled from the West ends of the IB (IBPw) and SIO (SIOPw) piers (all 5 sampling rounds (SR)). Tijuana River water was sampled from the Hollister Street bridge (TJRw; SR 2-5). See Fig. 1 for sampling locations. We sampled the mouth of the Tijuana River

on two occasions but do not use those data in the study because access was prohibitively difficult and because ocean influence was significant and variable there, changing with tides and surf. Sampling at the Hollister Street bridge avoided this problem and therefore better characterized the Tijuana River.

Aerosol Filter Sampling

Aerosol were sampled onto 47 mm QMA quartz fiber filters (Pall, Port Washington, NY) at 30 liters per minute. Quartz filters were precombusted at 500 °C for two hours and combusted filters were analyzed as blanks via mass spectrometry and 16S sequencing. Total suspended particles were collected, without any size exclusion, at ambient temperature and relative humidity.

Meteorological, Hydrological, and Oceanographic Data.

Wind data for IB were acquired from the KNRS meteorological station (32° 33' 47" N, 117° 6' 39" W), retrieved from weather.gladstonefamily.net. Wind data for LJ were acquired from cdip.ucsd.edu station 073 (SIO pier). Swell data were acquired from cdip.ucsd.edu. We use swell data from CDIP buoy 155 off IB because the primary purpose of the swell data is to determine if swell conditions were present that could have been driving alongshore currents. Elevated wave heights (>1 m) from the South or North can cause along shore currents that would transport pollution from the Tijuana River along the coast. Tijuana River flows at the international boundary (32° 32′ 36″ N, -117° 3′ 1″ W (WGS 84)) were acquired from the International Boundary and Water Commission at waterdata.ibwc.gov. Precipitation data for the locations of

KNKX (MCAS Miramar; 32° 51' 52" N, 117° 8' 12" W), KSDM (Brown Field; 32° 34' 20" N, 116° 58' 49" W), KCZZ (Campo; 32° 37' 34" N, 116° 28' 06" W), KNRS (IB; 32° 33' 47" N, 117° 6' 39" W), from mesowest.utah.edu [73,74]. Precipitation data at the Tijuana River estuary (32.57450, 117.12700) were acquired from the National Estuarine Research Reserve System (NERRS). Precipitation data for Los Penasquitos Lagoon (32° 56' 01" N, 117° 15' 26" W) were provided by J. McCullough and J. Crooks at the Tijuana River National Estuarine Research Reserve. Precipitation totals from KNKX (MCAS Miramar) and Los Penasquitos Lagoon were averaged for an estimate of precipitation in the LJ area. Precipitation data from KSDM, KCZZ, KNRS, and the Tijuana River estuary were averaged for an estimation of precipitation in the Tijuana River watershed. Aerosol particle volume was measured with the TSI 3321 aerodynamic particle sizer (TSI Inc, Shoreview, MN).

Sample Preparation Mass Spectrometry

Sea water samples were taken with a 20 L acid cleaned bucket (HomeDepot) at the water surface. 1 L of seawater was then transferred to a 1L HD-PP bottle and stored on ice till extraction in the laboratory (within 5 h from sampling). For solid phase extraction, the samples were acidified to pH 2 with ~ 1.2 mL hydrochloric acid (38% p.a.; trace metal grade, J.T. Baker, USA). The samples were then extracted through PPL cartridges with a bed mass of 200 mg. Before use, the cartridges were rinsed and activated with three cartridge volume of methanol (LC-MS grade, Fisher Scientific, San Diego, USA) and conditioned with two cartridge volumes of water (LC-MS grade, Fisher Scientific, San Diego, USA) at pH 2 (acidified with HCl, 37% p.a.; trace metal grade, J.T. Baker, USA). For extraction, acidified seawater was pulled through

the PPL cartridge with a flow rate below 10 ml/min with the help of a vacuum SPE station. Subsequently, remaining salt was removed with three cartridge volumes of pH 2 water. After drying with nitrogen gas, DOM was eluted with 2 ml of methanol into glass vials. After elution, the extracts were dried in a vacuum centrifuge (Centrivap, Labconco, Kansas City, USA) and stored at -80° C until further analysis. For LC-MS/MS analysis, samples were re-suspended in 100 µL MeOH/H2O/Formic acid (80/19/1).

Tandem Mass Spectrometry Analysis

UHPLC-MS/MS analysis was performed as described before [75,90]. In short, the extracted samples were re-dissolved in 100 µL methanol/water/formic acid (80:19:1, Fisher Scientific, San Diego, USA) of which 10 µL were injected into vanquish UHPLC system coupled to a Q-Exactive quadrupole orbitrap mass spectrometer (Thermo Fisher Scientific, Bremen, Germany) in two technical replicates. For the chromatographic separation, a reversed phase C18 porous core column (Kinetex C18, 150 x 2 mm, 1.8 um particle size, 100 A pore size, Phenomenex, Torrance, USA) was used. For gradient elution a high-pressure binary gradient system was used. The mobile phase consisted of solvent A H2O + 0.1 % formic acid (FA) and solvent B acetonitrile (ACN) + 0.1 % FA. The flow rate was set to 0.5 mL/min. After injection, the samples were eluted with a linear gradient from 0-0.5 min, 5 % B, 0.5-8 min 5-50 % B, 8-10 min 50-99 % B, followed by a 2 min washout phase at 99% B and a 3 min re-equilibration phase at 5 % B. Data dependent acquisition (DDA) of MS/MS spectra was performed in positive mode. Electrospray ionization (ESI) parameters were set to 52 L/min sheath gas flow, 14 L/min auxiliary gas flow, 0 L/min sweep gas flow and 400 °C auxiliary gas temperature. The spray

voltage was set to 3.5 kV and the inlet capillary to 320 °C. 50 V S-lens level was applied. MS scan range was set to 150-1500 m/z with a resolution at m/z 200 (Rm/z 200) of 70,000 with one micro-scan. The maximum ion injection time was set to 100 ms with automated gain control (AGC) target of 1.0E6. Up to 5 MS/MS spectra per MS1 survey scan were recorded DDA mode with Rm/z 200 of 17,500 with one micro-scan. The maximum ion injection time for MS/MS scans was set to 100 ms with a AGC target of 3.0E5 ions and minimum 5 % C-trap filling. The MS/MS precursor isolation window were set to m/z 1. Normalized collision energy was set to a stepwise increase from 20 to 30 to 40 % with z = 1 as default charge state. MS/MS scans were triggered at the apex of chromatographic peaks within 2 to 15 s from their first occurrence. Dynamic precursor exclusion was set to 5 s. Ions with unassigned charge states were excluded from MS/MS acquisition as well as isotope peaks.

Mass Spectrometry Data Analysis

For MS/MS data analysis raw spectra were converted to .mzXML files using MSconvert (ProteoWizard) MS1 and MS/MS feature extraction was performed using MZmine2.37 IIMN enabled version [76]. For peak picking an intensity threshold of 1E5 and for MS1 spectra and of 1E3 for MS/MS spectra was used. For MS1 chromatogram building a 5 ppm mass accuracy and a minimum peak intensity of 3E5 was set. Extracted Ion Chromatograms (XICs) were deconvoluted using the baseline cut-off algorithm with the baseline set to 1E5, a minimum peak intensity of 3E5 and a minimum peak duration of 0.01 min. After chromatographic deconvolution, MS1 features were linked to MS/MS spectra within 0.01 m/z mass and 0.2 min retention time windows. Isotope peaks were grouped using the isotope grouper module and

features from different samples were aligned with 5 ppm mass tolerance and 0.1 min retention time tolerance. MS1 features without MS2 features assigned were filtered out the resulting matrix as well as features which did not contain a minimum of 2 peak per isotope pattern and which did not occur at least in 2 samples. After filtering, gaps in the feature matrix were filled with the peak finder algorithm with a retention time tolerance of 0.1 min and 5 ppm mass tolerance. Finally, peak areas were exported in a feature table as .csv file and corresponding consensus MS/MS spectra as .mgf file. Contaminant features observed in the PPL process blanks with a relative peak area > 30% in comparison to the sample average were filtered out. For feature-based molecular networking and spectrum library matching the .mgf file was uploaded to GNPS (gnps.ucsd.edu) [77]. For spectrum library matching (against the GNPS and NIS17 library) and spectral networking the minimum cosine score to define spectral similarity was set to 0.7. The Precursor and Fragment Ion Mass Tolerances were set to 0.01 Da and Minimum Matched Fragment Ions to 4, Minimum Cluster Size to 1 (MS Cluster off). When Analog Search was performed the maximum mass difference was set to 100 Da. The GNPS library currently contains 74044 MS/MS spectra (Dec 18, 2019) including Mass Bank, ReSpect and HMDB. RPCA of MS1 data, normalized to total ion current (TIC) were then created with using Aitchinson distances and organic matter compositional distance (Bray-Curtis) was correlated against different meta-data categories with ADONIS (999 permutation) using Qiime2 [78].

Sample Preparation 16S Amplicon Sequencing

Ocean and river water samples were prepared on site by transferring 400µL into individual MoBio PowerSoil bead-beating tubes. Water samples and aerosol filters were stored at -80C

until further processing. When all study samples were collected, samples were plated using sterile technique and tools. A quarter of each aerosol filter sample was cut and placed into an individual MoBio PowerSoil bead-beating tube. To implement the KatharoSeq workflow for low biomass samples, each extraction plate contained a 10-fold serial dilution of ZymoBIOMICS™ Microbial Community Standard (D6300 Zymo, USA) ranging from 0.1 to 1 million cells per extraction. DNA was extracted using the Qiagen MagAttract PowerSoil DNA KF (384) Kit (Qiagen, Germany), following manufacturer's instructions. Briefly, 60µL of SL solution was added to each bead-beating tube [79]. Tubes were then beaten for 20 minutes at 20Hz using a TissueLyzer (Qiagen, Germany) and subsequently centrifuged at 3700xg for 5 mins. Lysates were transferred (400µL/tube) to a 96-deep well plate containing 450µL of IR Solution. After vortexing for seconds, plates were incubated at 4C for 10 mins and centrifuged at 3700xg for 5 mins. Supernatants (850µL/well) were transferred to a clean 96-deep well plate and centrifuged at 3700xg for 5 mins. Supernatants (450µL/well) were transferred to a 96-deep well plate containing 470µL of ClearMag Beads/ClearMag Binding Solution. This plate was further processed using the KingFisher Flex platform, using the protocol provided by the manufacturer. DNA was eluted in 65µL and stored at -20C until further processing.

Amplicon Sequencing

Extracted DNA was amplified in triplicate 10µL PCR reactions, containing 2µL of gDNA, and using the Earth Microbiome Project standard 16S 515f/806rB bar-coded primers [79,80]. Replicate reactions for each sample were pooled together, and 5µL per pool was combined for the final

library. Sequencing was performed on the Illumina MiSeq platform with 2x150bp paired-ends reads.

16S Amplicon Sequencing Data Analysis

Raw sequencing reads were demultiplexed, quality filtered and denoised using Deblur in QIITA under study ID 12758 [91,72]. Deblurred reads considered to be reagent contaminants were removed using the R package decontam [81]. Downstream data processing was performed using QIIME2 [78]. Serially diluted mock communities were used to assess the sequencing depth needed to pass the KatharoSeq workflow, so that 80% of positive controls reads correctly aligned to the mock communities' composition. RPCA ordination plots were obtained using DEICODE [82]. To assess the potential source of aerosol ASVs, SourceTracker2 was used setting ocean and river water samples as sources and aerosol samples as sinks .

Air Parcel Back Trajectories

A local particle origin for each sampling period was derived from local winds and running FLEXPART back trajectories initiated 24 hs prior to the initiation of each aerosol sampling period and terminating at the end of the sampling period (Fig. S2). We chose the period of 24 hs to capture short range transport of locally produced particles. Note particles from long range transport would also be present following those same trajectories. Simulated particles were released at 5 m elevation and allowed to travel horizontally and vertically, but we do not display the altitudes because we are only concerned with the horizontal (lat/lon) information (Fig. S2). Local particle origins were classified as from the sea or from the land when winds and back

trajectories agreed on either; otherwise, a "mixed" local particle origin was assigned. Air parcel back trajectories were carried out using the Flexible Particle Dispersion Model (FLEXPART), a Lagrangian transport and dispersion model [83]. The model was run using atmospheric circulation data from the National Centers for Environmental Prediction (NCEP) Climate Forecast System Reanalysis (CFSR) 6-hourly Products. For each aerosol sampling period, the simulation was run in back trajectory mode between the end of the sampling period and one day (24 h) prior to the start of the ~1d sampling period. Each FLEXPART run simulated the release of 500 air parcels from the sampling coordinates and height, with convection activated, and outputs were generated and averaged every hour.

Bacterial Counts

Heterotrophic bacteria counts from aerosols collected into solution with the Series 100 Universal Spot Sampler (model SS110A; Aerosol Devices Inc, Fort Collins, CO) were determined by flow cytometry at The Scripps Research Institute Flow Core facility. Sample preservation and cell staining followed Noble & Fuhrman [87]. Liquid samples were fixed with glutaraldehyde at a final concentration of 0.05%, incubated for 15 minutes at 5°C, then flash frozen with liquid nitrogen, and stored at 5°C. At the Flow Core, samples were thawed, diluted 1:10 in 1x TE buffer (pH 8), stained with SYBR Green I at a final dilution of 1:100 from the stock, and incubated for 10 minutes in the dark just before analysis by a Bio-Rad ZE5 Cell Analyzer. Heterotrophic bacteria were gated as a group in cytograms plotting fluorescence (488 nm laser; green fluorescence) against side scatter. Counts from this region from ultrapure water processed as a sample were subtracted as a blank.

Data not in manuscript but available at https://doi.org/10.6075/J07944V3

Total Organic Carbon

For total organic carbon (TOC) analysis, water was collected into combusted 40-mL borosilicate vials and sealed using acid-washed caps with septa. Samples were immediately acidified to pH 2 using trace metal grade 12N HCl(Fisher Scientific, San Diego, CA). Acidified samples were stored at room temperature in the dark until analyzed by high-temperature combustion with a Shimadzu 500 V-CSN/TNM-1 (Shimadzu Corp, Kyoto, Japan) that was modified by using a quartz column filled with platinum catalyst beads to improve peak shape. Combustion columns were pre-conditioned on 40-100 injections of 0.2 μm filtered seawater. A magnesium perchlorate water trap was used upstream of the halogen trap. A CO_2-free carrier gas was used to precondition the column, and ultra-high purity grade O_2 was delivered to the instrument as the carrier gas during sample analysis. Each acidified TOC sample was sparged for two minutes and measured following high temperature combustion at 680˚C. During analysis, five 100 μL injections were made from a single sample reservoir, and samples were reanalyzed when the %CV of the best 3 injections was >5%. The measurements were calibrated using an 8 point calibration curve between 10 and 100 μM C of potassium phthalate in Milli-Q water. Milli-Q water and a reference water sample were analyzed every 10 samples. Reference standards were obtained from NSF-supported deep Florida Strait (Batch 6FS–2006; Dennis Hansell, RSMAS, University of Miami). The expected concentration range for

Batch reference materials is provided at http://www.rsmas.miami.edu/groups/biogeochem/. Further details of sample analysis details and data verification protocols can be found at cce.lternet.edu/research.5758

Inorganic Nutrient Analysis

Nitrate, nitrite, phosphate, silicate, and ammonia concentrations in water samples were measured by the Oceanographic Data Facility (ODF) chemistry laboratory at Scripps Institution of Oceanography [84–86]. Following collection in the field, 30 ml water samples were kept cold and dark until stored frozen in the lab. Nutrient analyses were performed on a continuous-flow AutoAnalyzer (AA3; Seal Analytical, Mequon, WI). Standards at >97% purity were used from Johnson Matthey Chemical Co. (San Diego, CA) and Fisher Scientific (Waltham, MA).

Aerosol Counts

Aerosol counts (da = 0.5-20 um) were measured using an Aerodynamic Particle Sizer (APS, TSI Inc. Model 3321). Particles were not dried prior to measurement; thus, all data was collected at ambient relative humidity. The size distributions were converted to aerosol volume distributions and integrated to give the total aerosol volume (i.e. the total volume of all particles per volume of air), in the da = 0.5-20 um size range, assuming spherical particles. No density or shape factor

corrections were applied to the data, as these properties are unknown for the aerosol populations measured during the study.

Chlorophyll-a

Chlorophyll-a was measured on water samples according to the CalCOFI Chlorophyll Method [88]. Briefly, 50-250 ml of a water sample was filtered at <10 psi through a glass fiber filter. We diverged from the CalCOFI method, which specifies a Whatman GFF filter, in the filter we used: VWR 2833-129 Glass Fiber Filter, 47 mm diameter, grade 691, 1.5 um particle retention. As the primary target was large phytoplankton (>5 um diameter), we consider the divergence minor. To extract the chlorophyll, filters were placed in 8 ml of 90% acetone, shaken by hand, then sat overnight in the dark at 4°C. After extraction, the solutions were read for absorbance at 664 nm and 750 nm using a Turner 10AU fluorometer to determine chlorophyll-a concentration.

ACKNOWLEDGMENT

This project was supported by the Understanding and Protecting the Planet (UPP) initiative from the University of California San Diego and by the German Research Foundation (DFG) with Grant PE 2600/1 to DP. We thank for accommodating our research: the city of Imperial Beach and IB lifeguards, including Robert Stabenow, Jason Lindquist, Art Ayala, Trevor Spence, Adam Wraight, Jesus Gonzalez; the Tijuana River National Estuary Research Reserve and CA State

Parks, including Chris Peregrin, Jeff Crooks, Justin McCullough, Cara Stafford; CA Border Patrol Agents Amber Craig, Clinton Cox, and Paul Sheehan; UCSD-SIO: Monica Castrejón, Christian McDonald. We thank for research assistance: Kathryn Mayer, Gavin Cornwell, James Garrafa-Luna; TSRI: Brian Seegers, Alan Saluk. We thank three anonymous reviewers for their feedback. We acknowledge that the site of this study and UC San Diego lie in the stolen territory of the Kumeyaay people.

REFERENCES for Tables S1 & S2 and other Supporting Information

1. Zimmer-Faust, A. G.; Steele, J. A.; Xiong, X.; Staley, C.; Griffith, M.; Sadowsky, M. J.; Diaz, M.; Griffith, J. F. A Combined Digital PCR and Next Generation DNA-Sequencing Based Approach for Tracking Nearshore Pollutant Dynamics Along the Southwest United States/Mexico Border. *Front. Microbiol*. **2021**, 12, 674214.

2. Barboza, K.; Cubillo, Z.; Castro, E.; Redondo-Solano, M.; Fernandez-Jaramillo, H.; Arias Echandi, M. L. First isolation report of Arcobacter cryaerophilus from a human diarrhea sample in Costa Rica. *Rev. Inst. Med. Trop. Sao Paulo* **2017**, 59, e72.

3. Collado, L.; Figueras, M. J. Taxonomy, epidemiology, and clinical relevance of the genus Arcobacter. *Clin. Microbiol. Rev*. **2011**, 24, 174–192.

4. Numberger, D.; Ganzert, L.; Zoccarato, L.; Mühldorfer, K.; Sauer, S.; Grossart, H.-P.; Greenwood, A. D. Characterization of bacterial communities in wastewater with enhanced taxonomic resolution by full-length 16S rRNA sequencing. *Sci. Rep*. **2019**, 9, 9673.

5. Encyclopedia of Food Microbiology **2014**. doi:10.1016/c2009-1-61842-6.

6. Lowry, R.; Balboa, S.; Parker, J. L. & Shaw, J. G. Aeromonas flagella and colonisation mechanisms. *Adv. Microb. Physiol*. **2014**, 65, 203–256.

7. Wisplinghoff, H. Pseudomonas spp.; Acinetobacter spp. and Miscellaneous Gram-Negative Bacilli. In *Infectious Diseases*. Cohen, J.; Powderly, W. G.; Opal, S. M. Elsevier. **2017**, 1579–1599.

8. Guérin, C.; Lee, B.-H.; Fradet, B.; Dijk, E. v.; Mirauta, B.; Transcriptome architecture and regulation at environmental transitions in flavobacteria: the case of an important fish pathogen. *ISME Communications*. **2021**, 1.

9. Bergey's Manual of Systematics of Archaea and Bacteria, **2015**. doi:10.1002/9781118960608.

10. Pham, D. N.; Clark, L. & Li, M. Microplastics as hubs enriching antibiotic-resistant bacteria and pathogens in municipal activated sludge. *Journal of Hazardous Materials Letters* **2021**, 2, 100014.

11. Bergan, T. Human- and Animal-Pathogenic Members of the Genus Pseudomonas. *The Prokaryotes* **1981**, 666–700 doi:10.1007/978-3-662-13187-9_59.

12. Amann, R.; Ludwig, W.; Schulze, R.; Spring, S.; Moore, E.; Schleifer, K.-H. rRNA-Targeted Oligonucleotide Probes for the Identification of Genuine and Former Pseudomonads. *Systematic and Applied Microbiology* **1996**, 19, 501–509.

13. Kämpfer, P.; Schulz, R.; Jäckel, U.; Malik, A. K.; Aman, R.; Spring, S. Hydrogenophaga defluvii sp. nov. and Hydrogenophaga atypica sp. nov.; isolated from activated sludge. *Int. J. Syst. Evol. Microbiol*. **2005**, 55, 341–344.

14. Li, W.; Zheng, T.; Ma, Y.; Liu, J. Influences of flow conditions on bacterial communities in sewage and greywater small diameter gravity sewer biofilms. *Environ. Res*. **2020**, 183, 109289.

15. Wolff, D. Krah, D.; Dotsch, A.; Ghattas, A.-K.; Wick, A.; Ternes, T. A. Insights into the variability of microbial community composition and micropollutant degradation in diverse biological wastewater treatment systems. *Water Res*. **2018**, 143, 313–324.

16. Kalmbach, S. In situ probing reveals Aquabacterium commune as a widespread and highly abundant bacterial species in drinking water biofilms. *Water Res.* **2000**, 34 575–581.

17. Regalado, N. G.; Martin, G.; Antony, S. J. Acinetobacter lwoffii: bacteremia associated with acute gastroenteritis. *Travel Med. Infect. Dis*. **2009**, 7, 316–317.

18. Kozińska, A.; Paździor, E.; Pękala, A. & Niemczuk, W. Acinetobacter johnsonii and Acinetobacter lwoffii - the emerging fish pathogens. *Bulletin of the Veterinary Institute in Pulawy* **2014**, 58 193–199.

19. Lemaire, O. N.; Méjean, V. & Iobbi-Nivol, C. The Shewanella genus: ubiquitous organisms sustaining and preserving aquatic ecosystems. *FEMS Microbiol. Rev*. **2020**, 44, 155–170.

20. Vignier, N. Barreau, M.; Olive, C.; Baubion, B.; Theodose, R.; Hochedez, P.; Cabié, A. Human infection with Shewanella putrefaciens and S. algae: report of 16 cases in Martinique and review of the literature. *Am. J. Trop. Med. Hyg*. **2013**, 89, 151–156.

21. Jones, R. L. Clostridial Enterocolitis. *Veterinary Clinics of North America: Equine Practice*. 2000, 16 471–485.

22. Labus, J. S. , Osadchiy, V.; Hsiao, E. Y.; Tap, J.; Derrien, M.; Gupta, A.; Tillisch, K.; Le Nevé, B.; Grinsvall, C.; Ljungberg, M.; Öhman, L.; Törnbom, H.; Simren, M.; Mayer, E. A. Evidence for an association of gut microbial Clostridia with brain functional connectivity and gastrointestinal sensorimotor function in patients with irritable bowel syndrome, based on tripartite network analysis. *Microbiome* **2019**, 7, 45.

23. Borriello, S. P. Clostridial Disease of the Gut. *Clinical Infectious Diseases*. **1995**, 20 S242–S250.

24. Palleroni, N. J. Introduction to the Family Pseudomonadaceae. The Prokaryotes **1981**, 655–665 doi:10.1007/978-3-662-13187-9_58.

25. Bennett, K. W.; Eley, A. Fusobacteria: new taxonomy and related diseases. *J. Med. Microbiol*. **1993**, 39, 246–254.

26. Hong, P.-Y.; Li, X.; Yang, X.; Shinkai, T.; Zhang, Y.; Wang, X.; Mackie, R. I. Monitoring airborne biotic contaminants in the indoor environment of pig and poultry confinement buildings. *Environ. Microbiol*. **2012**, 14, 1420–1431.

27. Guiry, M. D.; Guiry, G. M.; Morrison, L.; Rindi, F.; Miranda, S. V.; Mathieson, A. C.; Parker, B. C.; Langangen, A.; John, D. M.; Barbara, I.; Carter, C. F.; Kuipers, P.; Garbary, D. J. AlgaeBase: An On-line Resource for Algae. *Cryptogamie, Algologie*. **2014**, 35(2), 105–115.

28. Vandepitte, L.; Vanhoorne, B.; Decock, W.; Vranken, S.; Lanssens, T.; Dekeyzer, S.; Verfaille, K.; Horton, T.; Kroh, A.; Hernandez, F.; Mees, J. A decade of the World Register of Marine Species - General insights and experiences from the Data Management Team: Where are we, what have we learned and how can we continue? *PLoS One.* **2018**, 13, e0194599.

29. Wexler, H. M. Bacteroides: the good, the bad, and the nitty-gritty. *Clin. Microbiol. Rev*. 2007, 20, 593–621.

30. Bernhard, A. E.; Field, K. G. A PCR assay to discriminate human and ruminant feces on the basis of host differences in Bacteroides-Prevotella genes encoding 16S rRNA. *Appl. Environ. Microbiol*. **2000**, 66, 4571–4574.

31. Ahmed, W.; Hughes, B.; Harwood, V. Current Status of Marker Genes of Bacteroides and Related Taxa for Identifying Sewage Pollution in Environmental Waters. *Water*. **2016**, 8, 231.

32. Baek, K. Jeon, C. O. Rheinheimera aestuari sp. nov.; a marine bacterium isolated from coastal sediment. *International Journal of Systematic and Evolutionary Microbiology*. **2015**, 65, 2640–2645.

33. Klein, A. N.; Frigon, D.; Raskin, L. Populations related to Alkanindiges, a novel genus containing obligate alkane degraders, are implicated in biological foaming in activated sludge systems. *Environ. Microbiol*. **2007**, 9, 1898–1912.

34. Guðmundsdóttir, R.; Kreiling, A.-K.; Kristjánsson, B. K.; Marteinsson, V. Þ.; Pálsson, S. Bacterial diversity in Icelandic cold spring sources and in relation to the groundwater amphipod Crangonyx islandicus. *PLoS One*. **2019**, 14, e0222527.

35. Erlacher, A.; Cardinale, M.; Grosch, R.; Grube, M.; Berg, G. The impact of the pathogen Rhizoctonia solani and its beneficial counterpart Bacillus amyloliquefaciens on the indigenous lettuce microbiome. *Front. Microbiol*. **2014**, 5, 175.

36. Carney, R. L.; Mitrovic, S. M.; Jeffries, T.; Westhorpe, D.; Curlevski, N.; Seymour, J. R. River bacterioplankton community responses to a high inflow event. *Aquatic Microbial Ecology*. **2015**, 75, 187–205.

37. Alfano, N.; Tagliapietra, V.; Rosso, F.; Manica, M.; Arnoldi, D.; Pindo, M.; Rizzoli, A. Changes in Microbiota Across Developmental Stages of Aedes koreicus, an Invasive Mosquito Vector in Europe: Indications for Microbiota-Based Control Strategies. *Frontiers in Microbiology*. **2019**, 10.

38. Padakandla, S. R.; Chae, J.-C. Reclassification of Aeromonas sharmana to a new genus as Pseudaeromonas sharmana gen. nov.; comb. nov.; and description of Pseudaeromonas pectinilytica sp. nov. isolated from a freshwater stream. *Int. J. Syst. Evol. Microbiol*. **2017**, 67, 1018–1023.

39. Wang, Z.; Li, W.; Li, H.; Zheng, W.; Guo, F. Phylogenomics of Rhodocyclales and its distribution in wastewater treatment systems. *Sci. Rep*. **2020**, 10, 3883.

40. Willems, A. The Family Comamonadaceae. The Prokaryotes **2014**, 777–851 doi:10.1007/978-3-642-30197-1_238.

41. Ueki, A. Paludibacter propionicigenes gen. nov.; sp. nov.; a novel strictly anaerobic, Gram-negative, propionate-producing bacterium isolated from plant residue in irrigated rice-field soil in Japan. *Int. J. Syst. Evol*. **2006**, 56, 39–44.

42. McLellan, S. L.; Huse, S. M.; Mueller-Spitz, S. R.; Andreishcheva, E. N.; Sogin, M. L. Diversity and population structure of sewage-derived microorganisms in wastewater treatment plant influent. *Environ. Microbiol*. **2010**, 12, 378–392.

43. van Esch, G. J.; World Health Organization & International Program on Chemical Safety. Flame Retardants: Tris(2-butoxyethyl) Phosphate, Tris(2-ethylhexyl) Phosphate and Tetrakis(hydroxymethyl) Phosphonium Salts **2000**.

44. Kim, S. Exploring Chemical Information in PubChem. *Curr. Protoc*. **2021**, 1, e217.

45. Pagel, H. A.; McLafferty, F. W. Use of Tributyl Phosphate for Extracting Organic Acids from Aqueous Solution. *Analytical Chemistry*. **1948**, 20, 272–272.

46. Davankov, V. and Tsyurupa, M. Sorption of Organic Compounds from Aqueous Solutions. in *Comprehensive Analytical Chemistry*. Elsevier, **2011**, 56, 411–444.

47. Marchetti, C.; Brown, A. M. Protein kinase activator 1-oleoyl-2-acetyl-sn-glycerol inhibits two types of calcium currents in GH3 cells. *Am. J. Physiol*. **1988**, 254, C206–10.

48. Sardar, S. W.; Choi, Y.; Park, N.; Jeon, J. Occurrence and Concentration of Chemical Additives in Consumer Products in Korea. *Int. J. Environ. Res. Public Health*, **2019**, 16.

49. Tasselli, S.; Guzzella, L. Polycyclic musk fragrances (PMFs) in wastewater and activated sludge: analytical protocol and application to a real case study. *Environ. Sci. Pollut. Res. Int*. **2020**, 27, 30977–30986.

50. Friedli, F. *Detergency of Specialty Surfactants*. CRC Press: Boca Raton, USA, **2001**.

51. Guedez, A. A.; Frömmel, S.; Diehl, P.; Püttmann, W. Occurrence and temporal variations of TMDD in the river Rhine, Germany. *Environ. Sci. Pollut. Res. Int*. **2010**, 17, 321–330.

52. Nagappayya, S. K.; Gaikar, V. G. Extraction of Aleuritic Acid from Seedlac and Purification by Reactive Adsorption on Functionalized Polymers. *Industrial & Engineering Chemistry Research*. **2010**, 49, 6547–6553.

53. Stone, D.; Ross, S. At Your Fingertips: Rapid Retrieval of Product Information from the National Pesticide Information Center. *Proceedings of the Vertebrate Pest Conference*. **2014**, 26.

54. den Hartigh, L. J. Conjugated Linoleic Acid Effects on Cancer, Obesity, and Atherosclerosis: A Review of Pre-Clinical and Human Trials with Current Perspectives. *Nutrients* **2019**, 11.

55. Ricciotti, E.; FitzGerald, G. A. Prostaglandins and Inflammation. *Arteriosclerosis, Thrombosis, and Vascular Biology* **2011**, 31, 986–1000

56. Takeshita, A.; Igarishi-Migitaka, J.; Takahashi, H.; Takeushi, Y.; Koibuchi, N. Acetyl tributyl citrate, the most widely used phthalate substitute plasticizer, induces cytochrome p450 3a through steroid and xenobiotic receptor. *Toxicol. Sci*. **2011**, 123, 460–470.

57. Triphenylphosphine oxide, 99%, Thermo ScientificTM. https://www.thermofisher.com/order/catalog/product/A12455.36.

58. Maresz, K. Proper Calcium Use: Vitamin K2 as a Promoter of Bone and Cardiovascular Health. *Integr. Med*. **2015**, 14, 34–39.

59. Amin, K. M.; Abou-Seri, S. M.; Awadallah, F. M.; Eissa, A. A. M.; Hassan, G. S.; Abdulla, M. M. Synthesis and anticancer activity of some 8-substituted-7-methoxy-2H-chromen-2-one derivatives toward hepatocellular carcinoma HepG2 cells. *Eur. J. Med. Chem*. **2015**, 90, 221–231.

60. Brose, S. A.; Thuen, B. T.; Golovko, M. Y. LC/MS/MS method for analysis of E2 series prostaglandins and isoprostanes. *Journal of Lipid Research*. **2011**, 52, 850–859.

61. Van Elssen, C. H. M. J.; Vanderlocht, J.; Oth, T.; Senden-Gijsbers, B. L. M. G.; Germeraad, W. T. V.; Bos, G. M. J. Inflammation restraining effects of prostaglandin E2 on natural killer–dendritic cell (NK-DC) interaction are imprinted during DC maturation. *Blood*. **2011**, 118, 2473–2482.

62. Ramanathan, R.; Chowdhury, S. K., Alton, K. B. Chapter 10 Oxidative metabolites of drugs and xenobiotics: LC-MS methods to identify and characterize in biological matrices. In *Identification and Quantification of Drugs, Metabolites and Metabolizing Enzymes by LC-MS.* **2005**, 225–276 doi:10.1016/s1464-3456(05)80012-2.

63. Duckworth, N.; Marshall, K.; Clayton, J. K. An investigation of the effect of the prostaglandin EP2 receptor agonist, butaprost, on the human isolated myometrium from pregnant and non-pregnant women. *J. Endocrinol* **2002**, 172, 263–269.

64. Consumer Product Safety Commission (CPSC). *Overview of Phthalates Toxicity*. U.S. CPSC, Bethesda, MD 20814. **2010**. https://www.cpsc.gov/s3fs-public/phthalover.pdf.

65. Schänzer, W.; Opfermann, G.; Donike, M. Metabolism of stanozolol: identification and synthesis of urinary metabolites. *J. Steroid Biochem*. **1990**, 36, 153–174.

66. Santa Cruz Biotechnology. 17-phenyl trinor Prostaglandin A2. https://www.scbt.com/p/17-phenyl-trinor-prostaglandin-a2-38315-51-4.

67. Knothe, G. Fuel Properties of Highly Polyunsaturated Fatty Acid Methyl Esters. Prediction of Fuel Properties of Algal Biodiesel. *Energy & Fuels*. **2012**, 26, 5265–5273.

68. Zempleni, J.; Mock, D. M. Biotin biochemistry and human requirements. *J. Nutr. Biochem*. **1999**, 10, 128–138.

69. Tsukamoto, S.; Kato, H.; Hirota, H.; Fusetani, N. Lumichrome. A larval metamorphosis-inducing substance in the ascidian Halocynthia roretzi. *European Journal of Biochemistry*. **1999**, 264, 785–789.

70. Sulistiyani, S.; Adelman, J.; Chandrasekaran, A.; Jayo, J.; St. Clair, R. W. Effect of 17α-Dihydroequilin Sulfate, a Conjugated Equine Estrogen, and Ethynylestradiol on Atherosclerosis in Cholesterol-Fed Rabbits. *Arteriosclerosis, Thrombosis, and Vascular Biology*. **1995**, 15, 837–846.

71. 13,14-dihydro-15-keto Prostaglandin F1α (CAS 29044-75-5). https://www.caymanchem.com/product/15670.

72. Gonzalez, A. et al. Qiita: rapid, web-enabled microbiome meta-analysis. *Nat. Methods*. **2018**, 15, 796–798.

73. Horel, J.; Splitt, M.; Dunn, L.; Pechmann, J.; White, B.; Ciliberti, C.; Lazarus, S.; Slemmer, J.; Zaff, D.; Burks, J. Mesowest: Cooperative Mesonets in the Western United States. *Bulletin of the American Meteorological Society*. **2002**, 83, 211–225.

74. Horel, J.; Potter, T.; Dunn, L.; Steenburgh, W. J.; Eubank, M.; Splitt, M.; Onton, D. J. Weather Support for the 2002 Winter Olympic and Paralympic Games. *Bulletin of the American Meteorological Society*. **2002**, 83, 227–240.

75. Petras, D. et al. High-Resolution Liquid Chromatography Tandem Mass Spectrometry Enables Large Scale Molecular Characterization of Dissolved Organic Matter. *Frontiers in Marine Science*. **2017**, 4.

76. Schmid, R. et al. Ion identity molecular networking for mass spectrometry-based metabolomics in the GNPS environment. *Nat. Commun*. **2021**, 12, 3832.

77. Nothias, L.-F. et al. Feature-based molecular networking in the GNPS analysis environment. *Nat. Methods*. **2020**, 17, 905–908.

78. Bolyen, E. et al. Reproducible, interactive, scalable and extensible microbiome data science using QIIME 2. *Nat. Biotechnol*. **2019** 37, 852–857.

79. Minich, J. J. et al. High-Throughput Miniaturized 16S rRNA Amplicon Library Preparation Reduces Costs while Preserving Microbiome Integrity. *mSystems*. **2018**, 3.

80. Thompson, L. R. et al. A communal catalogue reveals Earth's multiscale microbial diversity. *Nature*. **2017**, 551, 457–463.

81. Davis, N. M.; Proctor, D. M.; Holmes, S. P.; Relman, D. A.; Callahan, B. J. Simple statistical identification and removal of contaminant sequences in marker-gene and metagenomics data. *Microbiome* **2018**, 6, 1–14.

82. Martino, C. et al. A Novel Sparse Compositional Technique Reveals Microbial Perturbations. *mSystems* **2019**, 4.

83. Stohl, A.; Forster, C.; Frank, A.; Seibert, P.; Wotawa, G. Technical note: The Lagrangian particle dispersion model FLEXPART version 6.2. *Atmospheric Chemistry and Physics*. **2005**, 5, 2461–2474.

84. Hager, S. W.; E. L. ATLAS, Gordon, L. I.; Mantyla, A. W.; Park, P. K. A comparison at sea of manual and autoanalyzer analyses of phosphate, nitrate, and silicate1. *Limnol. Oceanogr*. **1972**, 17, 931–937.

85. Elliot L. Atlas, Hager, S. W.; Gordon, L. I.; Kilho Park, P. Oregon State University Corvallis Department of Oceanography. A Practical Manual for Use of the Technicon Autoanalyzer in Seawater Nutrient Analyses; Revised. **1971**.

86. Becker, S. et al. GO-SHIP Repeat Hydrography Nutrient Manual: The precise and accurate determination of dissolved inorganic nutrients in seawater, using Continuous Flow Analysis methods. **2019** doi:10.25607/OBP-555.

87. Noble, R. T. & Fuhrman, J. A. Use of SYBR Green I for Rapid Epifluorescence Counts of Marine Viruses and Bacteria. *Aquat. Microb. Ecol*. **1998**, 14, 113-118.

88. SIO-CalCOFI. Scripps Institution of Oceanography. California Cooperative Oceanic Fisheries Investigations Technical Group. Chlorophyll Methods. https://calcofi.org/references/methods/8-chlorophyll-methods.html.

89. Sousa, M. L. Assessing promising bioactivity of cyanobacterial strains on a 3D in vitro model of solid tumours. Mechanisms in physiologically relevant 3D cell culture. **2020**. https://repositorio-aberto.up.pt/bitstream/10216/126888/2/393006.pdf

90. Cancelada, L. et al. Assessment of styrene-divinylbenzene polymer (PPL) solid-phase extraction and non-targeted tandem mass spectrometry for the analysis of xenobiotics in seawater. *Limnology and Oceanography: Methods* **2022**, 20, 89–101.

91. Amir, A. et al. Deblur Rapidly Resolves Single-Nucleotide Community Sequence Patterns. *mSystems* **2017**, 2.

92. Gasol, J.M. and Del Giorgio, P.A. Using flow cytometry for counting natural planktonic bacteria and understanding the structure of planktonic bacterial communities, *Scientia Marina*. **2000**, 197–224. doi:10.3989/scimar.2000.64n2197.
